# Global, Regional, and National Burden of Pancreatic Cancer, 1990–2019: Results from the Global Burden of Disease Study 2019

**DOI:** 10.5334/aogh.4019

**Published:** 2023-05-25

**Authors:** Chengxia Kan, Na Liu, Kexin Zhang, Di Wu, Yunzi Liang, Weiqin Cai, Qi Jing, Fang Han, Shunjie Xing, Xiaodong Sun

**Affiliations:** 1 Department of Endocrinology and Metabolism, Affiliated Hospital of Weifang Medical University, Weifang, China; 2 Clinical Research Center, Affiliated Hospital of Weifang Medical University, Weifang, China; 3 Department of Pathology, Affiliated Hospital of Weifang Medical University, Weifang, China; 4 School of Management, Weifang Medical University, Weifang, China; 5 Department of ophthalmology, Affiliated Hospital of Weifang Medical University, Weifang, China; 6 Department of Endocrinology and Metabolism, Affiliated Hospital of Weifang Medical University, China

**Keywords:** Pancreatic cancer, Public health, incidence, risk factors

## Abstract

**Aims:**

Pancreatic cancer (PC) is a malignant tumor with a strong invasive nature and low survival rate. We aimed to estimate the PC burden at the global, regional, and national levels in 204 countries from 1990 to 2019.

**Methods:**

Detailed data, including the incidence, death, and disability-adjusted life years (DALYs), were analyzed from the Global Burden of Diseases Study 2019.

**Results:**

Globally, there were 530,297 (486,175–573,635) incident cases and 531,107 (491,948–566,537) deaths from PC in 2019. The age-standardized incidence rate (ASIR) was 6.6 (6–7.1), and the age-standardized mortality rate (ASMR) was 6.6 (6.1–7.1) per 100,000 person-years. PC caused 11,549,016 (10,777,405–12,338,912) DALYs, with an age-standardized rate of 139.6 (130.2–149.1) per 100,000 person-years. There were increases in estimated annual percentage changes (EAPCs) of ASIR (0.83; 0.78–0.87), ASMR (0.77; 0.73–0.81), and age-standardized DALYs rate (ASDR) (0.67; 0.63–0.71). The global number of incident cases increased by 168.7%, from 197,348 (188,604–203,971) to 530,297 (486,175–573,635); the number of deaths increased by 168.2% from 198,051 (189,329–204,763) to 531,107 (491,948–566,537); and total DALYs increased by 148.5% from 4,647,207 (4,465,440–4,812,129) to 11,549,016 (10,777,405–12,338,912). East Asia and China recorded the highest number of incident cases, deaths, and DALYs. The proportion of deaths was attributable to smoking (21.4%), elevated fasting glucose (9.1%), and high BMI (6%).

**Conclusions:**

Our study updated the epidemiological trends and risk factors for PC. PC remains a major hazard to the sustainability of health systems worldwide, with an increasing incidence rate and mortality from 1990 to 2019. More targeted strategies are required to prevent and treat PC.

## Background

Pancreatic cancer (PC) is a common lethal cancer that originates in the pancreas, an organ that lies behind the lower part of the stomach and produces digestive enzymes and hormones [1,2]. Due to the lack of efficient diagnosis methods and characteristic clinical signs, PC patients often are diagnosed at a late stage and have local progression or distant metastasis on initial diagnosis [3]. Therefore, many patients lose the chance of radical surgery. The 2020 Global Cancer Registry data showed that PC ranked twelfth in cancer incidence rate and seventh in mortality rate [4]. In 2020, approximately 466,000 related deaths were recorded, accounting for approximately 4.7% of malignant tumor deaths. The number of incident cases and deaths are the highest in Asia (47.1% and 48.1%, respectively), followed by those in Europe (28.3% and 28.4%, respectively) and North America (12.6% and 11.4%, respectively) [5].

Mortality rates also vary widely among countries. Conversely, the mortality rates are very low in developing countries, such as in Africa and South Asia. These findings indicate that the PC burden in developed countries is higher than that in undeveloped countries [6]. The Global Burden of Disease (GBD) study includes crucial information on PC burden in 195 countries worldwide [7]. Thus, the study aimed to determine PC burden at the global, regional, and national levels in 204 countries from 1990 to 2019. Additionally, data were stratified by sex, region, and sociodemographic index (SDI), and risk factors for the proportion of disability-adjusted life years (DALYs, calculated as the sum of years of life lost because of disease and the number of years of life affected by the disease) were further analyzed.

## Methods

### Study data

Annual data on PC deaths, incidence, and DALYs stratified from 1990 to 2019 by region and sex were collected from the Global Health Data Exchange (GHDx) query tool (http://ghdx.healthdata.org/gbd-results-tool). Data available for 204 countries and territories were collected and categorized into five regions according to SDI. Also collected were human development index (HDI) data from the Human Development Reports (https://hdr.undp.org//en/composite/HDI).

### Statistical analysis

The age-standardized incidence rate (ASIR), age-standardized mortality rate (ASMR), age-standardized DALYs rate (ASDR), and estimated annual percentage change (EAPC) were used to quantify PC trends globally [8,9,10]. The direct method was applied to calculate age-standardized rates (ASRs), and the ASRs of PC per 100,000 population were fitted to the world standard population. The EAPC was calculated as 100 × (exp(β)-1), with its 95% CI from the linear regression model. An increasing ASR trend was defined as an EAPC estimation and minimum lower limit of the 95% CI being both > 0. Conversely, decreased ASR was defined as an EAPC estimation and maximum upper limit of the 95% CI both being < 0. The SDI reflects the social and economic conditions of a location with higher values indicating a higher development level. We examined the factors influencing EAPCs by assessing the correlation of EAPCs with ASRs (1990) and HDI (2019) at the national level. We performed all statistical analyses using R-Studio version 4.1.2 and set statistical significance at *p* < 0.05.

## Results

### Global burden of pancreatic cancer

**Figure 1:**
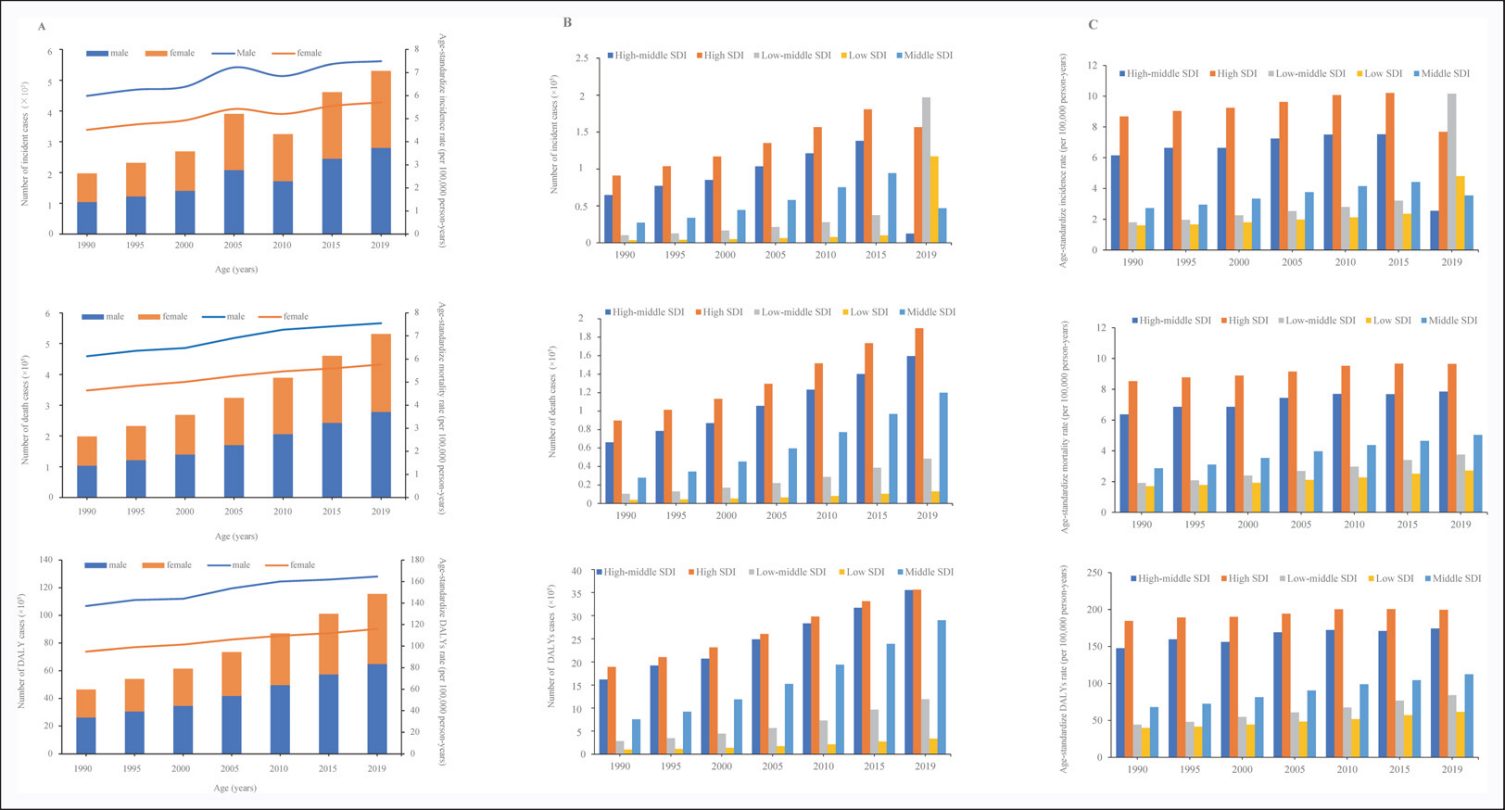
The number cases and ASRs of pancreatic cancer from 1990 to 2019. **(A)** The incidence, death and DALYs cases and rate of pancreatic cancer caused by global; **(B)** The incidence, death and DALYs cases and rate of pancreatic cancer caused by SDI regions; **(C)** The ASIR, ASMR, and ASDR of pancreatic cancer. ASIR, Age-standardized incidence rate; ASMR, age-standardized mortality rate; ASDR, age-standardized DALYs rate.

In 2019, there were 530,297 (486,175–573,635) incident cases of PC worldwide, which was a 168.7% increase from 197,348 (188,604–203,971) in 1990. The global ASIR also increased by 26.9% from 5.2 (5–5.4) in 1990 to 6.6 (6–7.1) in 2019. Likewise, the mortality for both sexes rose by 168.2% from 198,051 (189,329–204,763) in 1990 to 531,107 (491,948–566,537) in 2019. The global ASMR of PC was 5.3 (5.1–5.5) per 100,000 population in 1990, and this increased to 6.6 (6.1–7.1) in 2019. PC caused 11,549,016 (10,777,405–12,338,912) DALYs in 2019, and this was a 148.5% increase from 4,647,207 (4,465,440–4,812,129) in 1990. The ASDR showed an increase of 20.9%, from 115.5 (110.8–119.6) in 1990 to 139.6 (130.2–149.1) in 2019 (Table 1; Figure 1A).

**Table 1:** Incident cases, deaths, and DALYs of Pancreatic Cancer and ASRs per 100,00 population from 1990 and 2019 by Global Burden of Disease.


CHARACTERISTICS	1990		2019		1990–2019
		
	INCIDENT CASES	ASIR PER 100,000	INCIDENT CASES	ASIR PER 100,000	EAPC
		
	NO. (95% UI)	NO. (95% UI)	NO. (95% UI)	NO. (95% UI)	NO. (95% CI)

**Overall**	197,348 (188,604–203,971)	5.2 (5–5.4)	530,297 (486,175–573,635)	6.6 (6–7.1)	0.83 (0.78–0.87)

**Sex**					

Male	104,052 (99,198–108,991)	6 (5.7–6.3)	279,903 (256,009–303,427)	7.5 (6.8–8.1)	0.84 (0.79–0.9)

Female	93,296 (88,534–96,902)	4.5 (4.3–4.7)	250,393 (223,820–275,352)	5.7 (5.1–6.3)	0.81 (0.77–0.85)

**Socio-demographic index**					

Low	3632 (2909–4342)	1.6 (1.3–1.9)	12,561 (11,018–14,176)	2.5 (2.2–2.9)	1.67 (1.62–1.72)

Low-middle	10,304 (8852–11,799)	1.8 (1.5–2)	46,939 (42,996–51,039)	3.5 (3.2–3.8)	2.4 (2.36–2.45)

Middle	27,405 (25,465–29,352)	2.7 (2.5–2.9)	117,095 (104,632–130,584)	4.8 (4.3–5.3)	2.03 (1.97–2.1)

High-middle	64,779 (62,374–67,157)	6.1 (5.9–6.4)	156,544 (142,579–170,422)	7.7 (7–8.3)	0.74 (0.66–0.83)

High	91,158 (87,225–93,124)	8.7 (8.3–8.9)	196,919 (174,831–215,526)	10.2 (9.1–11.1)	0.62 (0.58–0.67)

**Regions**					

Andean Latin America	395 (349–445)	2 (1.7–2.2)	2870 (2344–3463)	5.2 (4.3–6.3)	3.64 (3.01–4.27)

Australasia	1823 (1740–1887)	7.8 (7.4–8)	4427 (3566–5396)	8.7 (7–10.7)	0.45 (0.38–0.52)

Caribbean	417 (396–437)	1.6 (1.5–1.7)	2649 (2216–3090)	5.1 (4.3–6)	3.84 (3.05–4.63)

Central Asia	1157 (1027–1295)	2.5 (2.2–2.8)	4116 (3761–4529)	5.8 (5.3–6.4)	3.66 (3.27–4.04)

Central Europe	12,294 (11,948–12,568)	8.3 (8.1–8.5)	22,048 (19,435–24,834)	10.3 (9.1–11.7)	0.79 (0.71–0.87)

Central Latin America	3527 (3403–3621)	4.4 (4.2–4.5)	12,504 (10,826–14,378)	5.4 (4.6–6.2)	0.45 (0.33–0.56)

Central Sub-Saharan Africa	477 (384–597)	2.2 (1.7–2.7)	1403 (1106–1769)	2.7 (2.2–3.4)	0.54 (0.26–0.82)

East Asia	27,929 (24,282–31,467)	3.2 (2.8–3.6)	119,571 (102,244–138,482)	5.8 (5–6.7)	2.31 (2.13–2.5)

Eastern Europe	18,854 (17,939–20,052)	6.7 (6.4–7.1)	27,330 (24,669–30,295)	8 (7.2–8.8)	0.28 (0.04–0.52)

Eastern Sub-Saharan Africa	1385 (1157–1634)	1.9 (1.6–2.2)	4460 (3755–5247)	2.8 (2.4–3.3)	1.41 (1.35–1.47)

High-income Asia Pacific	18,798 (17,948–19,289)	9.5 (9–9.8)	49,450 (41,236–56,372)	10.2 (8.7–11.7)	0.37 (0.27–0.48)

High-income North America	31,874 (30,346–32,741)	9 (8.6–9.2)	65,345 (57,314–74,242)	10.3 (9.1–11.7)	0.5 (0.47–0.53)

North Africa and Middle East	4519 (3841–5331)	2.7 (2.3–3.2)	22,237 (19,273–25,716)	5.3 (4.6–6.1)	2.5 (2.38–2.62)

Oceania	50 (40–64)	1.8 (1.4–2.2)	166 (133–209)	2.5 (2–3.1)	1.09 (0.97–1.21)

South Asia	7582 (6136–8936)	1.4 (1.1–1.7)	38,731 (34,043–43,732)	2.9 (2.5–3.2)	2.45 (2.38–2.53)

Southeast Asia	5951 (5439–6512)	2.4 (2.2–2.6)	25,376 (20,339–31,451)	4.3 (3.5–5.3)	2.04 (2–2.08)

Southern Latin America	3945 (3652–4247)	8.6 (8–9.3)	8841 (6977–11,035)	10.5 (8.3–13.1)	0.55 (0.4–0.7)

Southern Sub-Saharan Africa	1170 (1017–1399)	4.4 (3.8–5.3)	3303 (2984–3657)	6.1 (5.5–6.7)	0.93 (0.63–1.22)

Tropical Latin America	4477 (4307–4624)	5.1 (4.9–5.3)	14,752 (13,632–15,617)	6.2 (5.7–6.5)	0.73 (0.68–0.78)

Western Europe	48,837 (46,858–49,902)	8.4 (8.1–8.6)	92,785 (80,325–105,234)	10 (8.7–11.4)	0.69 (0.61–0.78)

Western Sub-Saharan Africa	1886 (1556–2244)	2.2 (1.9–2.6)	7932 (6509–9408)	4.5 (3.8–5.3)	2.44 (2.39–2.5)

**CHARACTERISTICS**	**1990**		**2019**		**1990–2019**
		
	**DEATHS CASES**	**ASMR PER 100,000**	**DEATHS CASES**	**ASMR PER 100,000**	**EAPC**
		
	**NO. (95% UI)**	**NO. (95% UI)**	**NO. (95% UI)**	**NO. (95% UI)**	**NO. (95% CI)**

**Overall**	198,051 (189,329–204,763)	5.3 (5.1–5.5)	531,107 (491,948–566,537)	6.6 (6.1–7.1)	0.77 (0.73–0.81)

**Sex**					

Male	103,312 (98,381–108,764)	6.1 (5.8–6.4)	278,174 (257,505–298,745)	7.5 (7–8.1)	0.79 (0.74–0.85)

Female	94,739 (89,322–98,184)	4.6 (4.3–4.8)	252,934 (225,846–273,820)	5.8 (5.1–6.2)	0.74 (0.71–0.77)

**Socio-demographic index**					

Low	3732 (2987–4466)	1.7 (1.4–2)	12,946 (11,336–14,669)	2.7 (2.4–3.1)	1.66 (1.61–1.71)

Low-middle	10,534 (8989–11,991)	1.9 (1.6–2.2)	48,532 (44,310–53,080)	3.8 (3.4–4.1)	2.38 (2.34–2.43)

Middle	27,840 (25,926–29,775)	2.9 (2.7–3.1)	120,021 (107,034–134,529)	5 (4.5–5.6)	2 (1.93–2.07)

High-middle	66,079 (63,329–68,574)	6.4 (6.1–6.6)	159,583 (146,077–170,902)	7.8 (7.2–8.4)	0.69 (0.61–0.77)

High	89,795 (85,585–91,855)	8.5 (8.1–8.7)	189,782 (171,237–200,955)	9.6 (8.8–10.2)	0.49 (0.45–0.53)

**Regions**					

Andean Latin America	412 (365–463)	2.1 (1.9–2.4)	3007 (2470–3615)	5.5 (4.5–6.6)	3.61 (2.97–4.24)

Australasia	1760 (1671–1822)	7.5 (7.1–7.8)	4158 (3736–4560)	8.1 (7.4–8.9)	0.32 (0.24–0.4)

Caribbean	433 (410–453)	1.7 (1.6–1.8)	2741 (2302–3188)	5.3 (4.4–6.2)	3.78 (2.99–4.58)

Central Asia	1174 (1039–1321)	2.6 (2.3–2.9)	4144 (3780–4556)	6.1 (5.5–6.7)	3.69 (3.3–4.07)

Central Europe	12,578 (12,196–12,873)	8.6 (8.3–8.8)	22,801 (19,902–25,677)	10.6 (9.3–11.9)	0.75 (0.67–0.83)

Central Latin America	3642 (3508–3742)	4.6 (4.4–4.8)	12,898 (11,082–14,848)	5.6 (4.8–6.4)	0.38 (0.27–0.5)

Central Sub-Saharan Africa	486 (397–603)	2.3 (1.9–2.8)	1424 (1129–1786)	2.9 (2.4–3.6)	0.52 (0.23–0.8)

East Asia	28,252 (24,692–32,012)	3.3 (3–3.8)	122,002 (104,214–140,812)	6 (5.1–6.9)	2.25 (2.07–2.43)

Eastern Europe	19,237 (18,286–20,441)	6.9 (6.5–7.3)	28,129 (25,268–31,110)	8.2 (7.3–9.1)	0.28 (0.05–0.51)

Eastern Sub-Saharan Africa	1428 (1197–1679)	2 (1.7–2.4)	4636 (3921–5448)	3.1 (2.6–3.6)	1.47 (1.4–1.54)

High-income Asia Pacific	17,677 (16,887–18,130)	9 (8.6–9.3)	45,214 (38,768–48,954)	9.2 (8.2–9.9)	0.16 (0.06–0.25)

High-income North America	31,377 (29,759–32,260)	8.8 (8.4–9)	63,557 (59,249–66,551)	9.9 (9.3–10.3)	0.44 (0.41–0.47)

North Africa and Middle East	4594 (3889–5424)	2.8 (2.4–3.4)	22,277 (19,357–25,691)	5.5 (4.8–6.3)	2.42 (2.25–2.59)

Oceania	51 (40–65)	1.9 (1.5–2.4)	167 (134–209)	2.6 (2.2–3.2)	1.08 (0.96–1.19)

South Asia	7736 (6243–9125)	1.5 (1.2–1.8)	40,012 (35,017–45,582)	3 (2.7–3.5)	2.41 (2.34–2.48)

Southeast Asia	6067 (5546–6595)	2.5 (2.3–2.7)	26,038 (20,861–32,271)	4.6 (3.6–5.7)	2.04 (2–2.08)

Southern Latin America	4114 (3795–4446)	9.1 (8.4–9.8)	9199 (8438–9924)	10.9 (10–11.7)	0.49 (0.34–0.63)

Southern Sub-Saharan Africa	1205 (1037–1437)	4.7 (4–5.6)	3411 (3078–3764)	6.5 (5.9–7.2)	0.96 (0.68–1.24)

Tropical Latin America	4601 (4399–4752)	5.4 (5.1–5.6)	15,313 (14,138–16,225)	6.4 (5.9–6.8)	0.69 (0.63–0.74)

Western Europe	49,267 (47,112–50,393)	8.4 (8–8.6)	91,771 (83,319–97,811)	9.7 (8.9–10.2)	0.56 (0.5–0.61)

Western Sub-Saharan Africa	1962 (1645–2306)	2.4 (2–2.8)	8209 (6811–9708)	4.9 (4.1–5.7)	2.45 (2.4–2.5)

**CHARACTERISTICS**	**1990**		**2019**		**1990–2019**	
		
	**DALYS CASES**	**ASDR PER 100,000**	**DALYS CASES**	**ASDR PER 100,000**	**EAPC**
		
	**NO. (95% UI)**	**NO. (95% UI)**	**NO. (95% UI)**	**NO. (95% UI)**	**NO. (95% CI)**

**Overall**	4,647,207 (4,465,440–4,812,129)	115.5 (110.8–119.6)	11,549,016 (10,777,405–12,338,912)	139.6 (130.2–149.1)	0.67 (0.63–0.71)

**Sex**					

Male	2,622,005 (2,488,009–2,776,580)	137.3 (130.5–144.9)	6,484,586 (5,984,574–6,995,914)	164.7 (151.9–177.4)	0.68 (0.62–0.73)

Female	2,025,202 (1,934,140–2,102,632)	95 (90.6–98.5)	5,0644,29 (4,630,521–5,462,302)	116 (106–125.1)	0.66 (0.63–0.68)

**Socio-demographic index**					

Low	100,183 (80,547–120,048)	39.7 (31.8–47.5)	335,996 (293,809–382,001)	61.4 (53.9–69.7)	1.55 (1.49–1.6)

Low-middle	280,954 (240,632–321,228)	44.2 (37.7–50.4)	1,188,693 (1,080,191–1,309,276)	84.2 (76.7–92.5)	2.29 (2.25–2.33)

Middle	753,881 (697,543–811,889)	67.9 (63.1–72.8)	2,901,059 (2,595,114–3,249,069)	112.5 (100.6–126)	1.81 (1.75–1.87)

High-middle	1,618,722 (1,552,786–1,680,981)	147.6 (141.6–153.3)	3,553,955 (3,272,690–3,819,144)	174.4 (160.4–187.4)	0.51 (0.42–0.6)

High	1,891,864 (1,832,587–1,922,820)	184.5 (179–187.4)	3,564,050 (3,347,923–3,733,175)	199.6 (188.7–208.4)	0.32 (0.28–0.35)

**Regions**					

Andean Latin America	10,198 (8982–11,509)	48 (42.3–54.1)	66,402 (53,656–80,688)	117.5 (95.2–142.4)	3.37 (2.77–3.98)

Australasia	37,286 (35,951–38,447)	159.5 (153.7–164.4)	78,744 (72,376–85,204)	166.8 (153.8–180.2)	0.22 (0.13–0.3)

Caribbean	10,060 (9583–10,536)	38.2 (36.4–39.9)	60,169 (50,197–70,456)	116 (96.7–135.6)	3.74 (2.97–4.51)

Central Asia	30,118 (27,058–33,497)	62 (55.4–69.2)	107,574 (97,906–118,737)	137.9 (125.8–151.8)	3.46 (3.06–3.86)

Central Europe	304,303 (297,472–310,263)	204.8 (200–208.9)	489,691 (428,369–552,784)	242 (211.1–273.7)	0.6 (0.52–0.68)

Central Latin America	89,927 (87,650–92,047)	103.2 (100.3–105.9)	296,170 (255,205–343,366)	123.6 (106.8–143.3)	0.37 (0.26–0.48)

Central Sub-Saharan Africa	13,594 (10,913–17,110)	54.7 (44.7–67.8)	39,510 (30,534–50,482)	67.7 (53.6–85.2)	0.46 (0.18–0.73)

East Asia	781,223 (674,711–895,330)	81.8 (71.1–93.2)	2,912,678 (2,471,047–3,375,822)	137 (116.5–158.7)	2.01 (1.84–2.18)

Eastern Europe	491,781 (468,431–523,367)	173.9 (165.6–185.3)	666,519 (598,990–741,493)	200.9 (180.3–223.3)	0.07 (–0.2–0.34)

Eastern Sub-Saharan Africa	38,360 (31,928–45,128)	47.7 (39.9–56)	123,529 (103,041–147,354)	70.3 (59.2–82.9)	1.37 (1.31–1.43)

High-income Asia Pacific	392,978 (380,803–400,893)	192 (185.6–196)	762,660 (686,247–808,974)	183.5 (169–192.9)	–0.11 (–0.2––0.01)

High-income North America	653,052 (632,123–666,614)	191.9 (186.3–195.7)	1,263,073 (1,201,537–1,309,882)	208.7 (199.4–216.1)	0.29 (0.27–0.32)

North Africa and Middle East	122,095 (104,334–144,551)	66.5 (56.7–78.7)	563,612 (484,686–654,879)	123 (106.4–142.5)	2.21 (2.05–2.37)

Oceania	1,410 (1,116–1,808)	43.8 (34.8–55.7)	4,604 (3,628–5,874)	60.3 (48.4–75.8)	1.03 (0.9–1.16)

South Asia	207,394 (169,063–244,804)	34.6 (27.9–40.8)	970,655 (854,924–1,107,519)	67.1 (59–76.4)	2.35 (2.24–2.45)

Southeast Asia	165,650 (150,118–181,571)	59.7 (54.3–65)	646,986 (519,553–799,457)	101.9 (81.8–126.1)	1.76 (1.71–1.82)

Southern Latin America	92,630 (86,352–99,146)	198.7 (185.4–212.7)	189,704 (175,665–203,127)	230.6 (214.1–246.9)	0.35 (0.2–0.5)

Southern Sub-Saharan Africa	30,733 (26,892–35,882)	106.6 (92.4–125.9)	83,713 (75,199–93,014)	143.3 (128.9–159.2)	0.84 (0.54–1.13)

Tropical Latin America	115,708 (111,570–119,204)	121.2 (116.5–124.9)	347,279 (325,679–365,771)	141.1 (132.1–148.8)	0.6 (0.55–0.64)

Western Europe	1,009,116 (980,031–1,026,588)	181 (176.2–184.1)	1,668,725 (1,5594,86–1,757,416)	199.5 (188–209.3)	0.41 (0.34–0.47)

Western Sub-Saharan Africa	49,592 (41,432–59,036)	54.2 (45.3–64.1)	207,020 (170,260–247,579)	105.8 (87.5–125.8)	2.31 (2.26–2.36)


ASIR, age-standardized incidence rate; ASMR, age-standardized mortality rate; ASDR, age-standardized DALYs rate; EAPC, estimated annual percentage change; CI, confidence interval; UI, uncertainty interval.

### Regional burden of pancreatic cancer

The number of PC cases increased in all five SDI regions (Figure 1B), with the low-middle region having the highest EAPC of ASIR (2.4; 95% CI: 2.36–2.45), ASMR (2.38; 2.34–2.43), and ASDR (2.29; 2.25–2.33) (Figure 1C, **Suppl Figure 1**). The ASIR and ASMR of PC in Southern Latin America were 10.5 (8.3–13.1) and 10.9 (10–11.7), which were the highest among the 21 GBD regions in 2019 (**Suppl Figure 2A&B**). Additionally, the ASDR in Central Europe was 242 (211.1–273.7), ranking first in 2019 (**Suppl Figure 2C**). The ASIR, ASMR, and ASDR of PC all increased in 21 regions, with the most significant being in the Caribbean (EAPC: 3.84 (3.05–4.63), 3.78 (2.99–4.58), and 3.74 (2.97–4.51), followed by that in Central Asia (EAPC: 3.66 (3.27–4.04), 3.69 (3.3–4.07), and 3.46 (3.06–3.86) and Andean Latin America (EAPC: 3.64 (3.01–4.27), 3.61 (2.97–4.24), and 3.37 (2.77–3.98) during the past 30 years (**Suppl Figure 3**).

### National burden of pancreatic cancer

**Figure 2:**
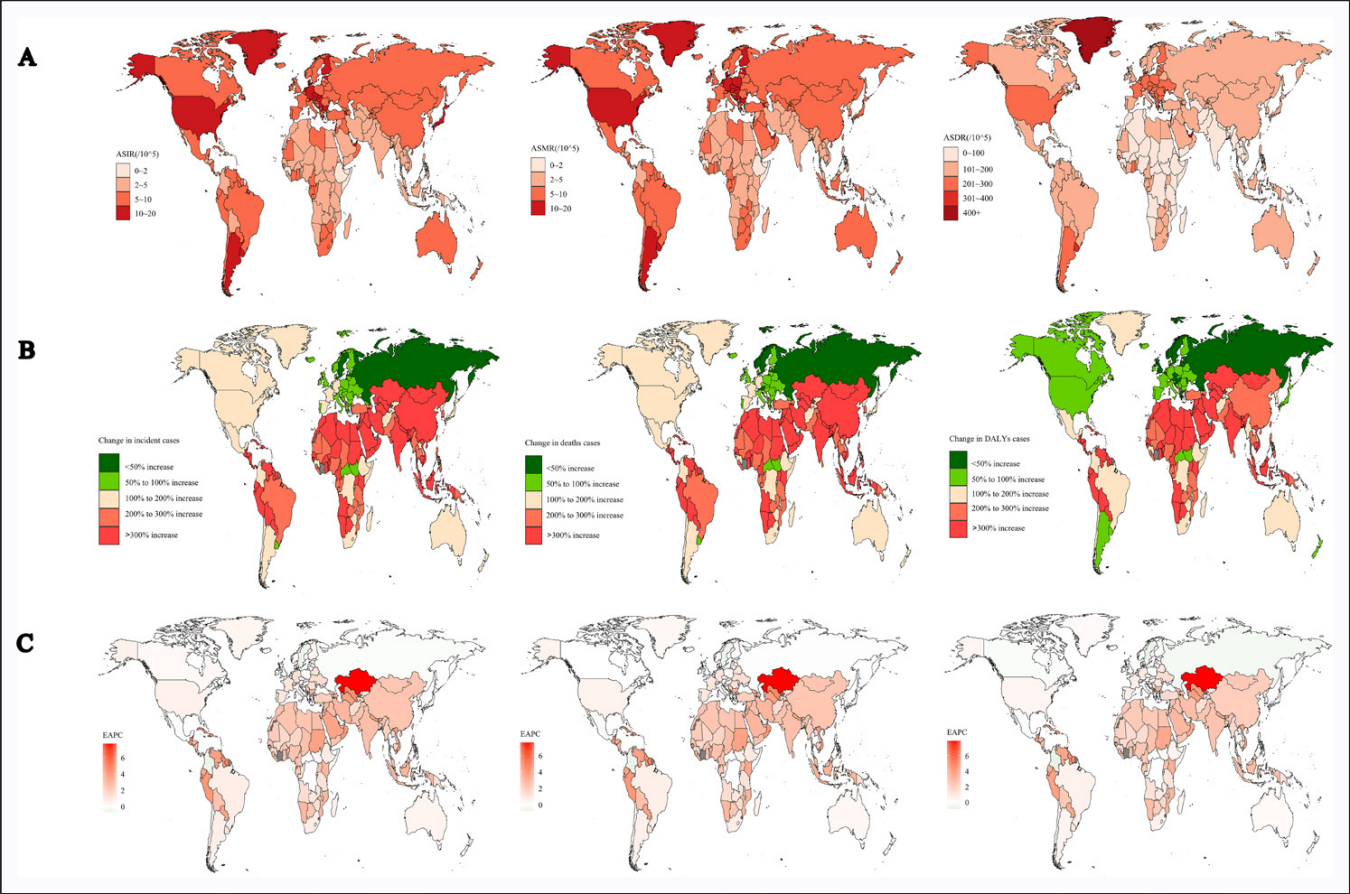
The national burden of pancreatic cancer for both sexes in 204 countries and territories. **(A)** The ASIR, ASMR, and ASDR of pancreatic cancer in 2019; **(B)** The relative change in incidence, deaths, and DALYs cases of pancreatic cancer between 1990 and 2019; **(C)** The EAPC of pancreatic cancer ADIR, ASMR, and ASDR from 1990 to 2019. ASIR, Age-standardized incidence rate; ASMR, age-standardized mortality rate; ASDR, age-standardized DALYs rate; EAPC, estimated annual percentage change.

Globally, China showed the highest rates at 114,964.2 (98,047.5–133,708.1) incident cases, 117,374 (99,862.7–136,452.7) mortalities, and 2,805,177.7 (2,368,769.3–3,276,551.5) DALYs. This comprised nearly 20% of the global totals in 2019. Greenland had the highest ASIR (18.9 [15.5–22.3]), ASMR (19.3 [15.7–22.8]), and ASDR (429.2 [345–517.4]) per 100,000 population in 2019. Conversely, Ethiopia displayed the lowest ASIR (1.5 (1–2.1)), ASMR (1.6 (1.1–2.3)), and ASDR (34.6 (23.1–50.2)) per 100,000 population (Figure 2A). The most pronounced rise was observed in the United Arab Emirates, with the incidence increasing by 2118.9%; deaths, 2050.2%; and DALYs, 2201.2% (Figure 2B). Kazakhstan showed the largest increase in ASIR (EAPC, 7.7 (6.5–8.8)), ASMR (EAPC, 7.5 (6.4–8.6)), and ASDR (EAPC, 7.8 (6.6–9.1)), followed by Cabo Verde and Grenada (Figure 2C; **Table S1**).

### Influencing factors of EAPC

**Figure 3:**
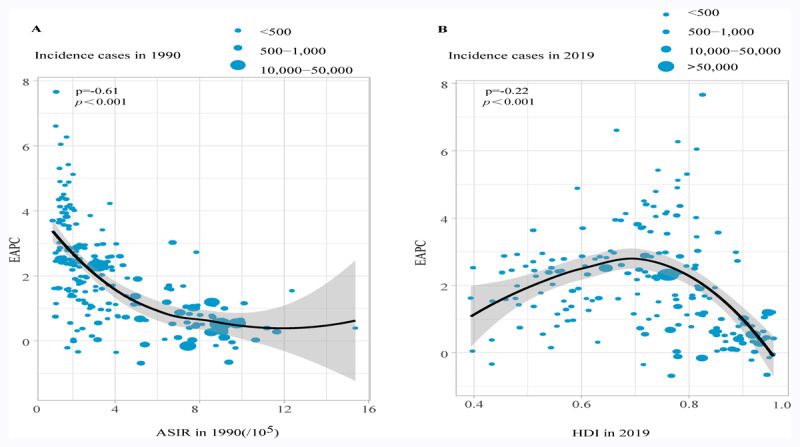
The correlation between EAPC and pancreatic cancer ASIR in 1990 and HDI in 2019. EAPC, estimated annual percentage change; ASIR, Age-standardized incidence rate; HDI, human development index.

EAPC were remarkably different from ASIR in 1990 and HDI in 2019 (Figure 3). The ASIR in 1990 represents the disease pool at baseline, and HDI reflects the level of medical care. EAPC was significantly negatively related to ASIR (*r =* –0.61, *P* < 0.001). Interestingly, when we restricted HDI to values below 0.7, we observed a positive correlation between EAPCs and HDI (*r =* –0.23, *P* = 0.007). In contrast, for HDI values above 0.7, EAPC had a significant negative association with HDI. (*r* = –0.23, *P* = 0.002).

### Association of ASRs with SDI

**Figure 4:**
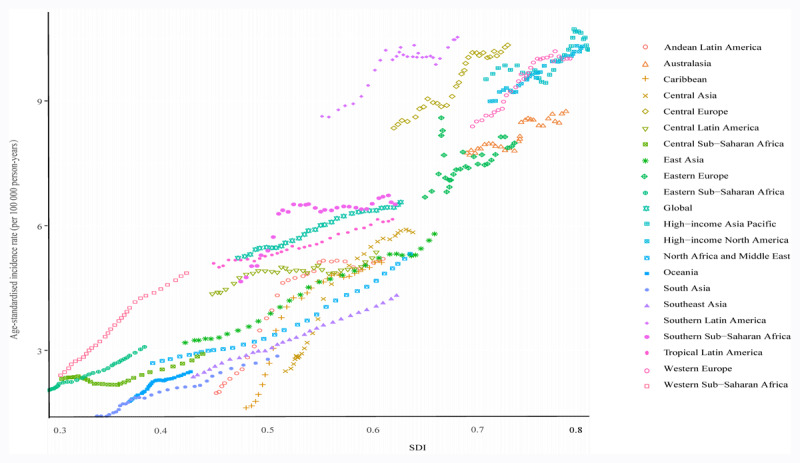
ASIR of pancreatic cancer in global and 21 regions by SDI, 1990–2019. ASIR, age-standardized incidence rate; SDI, sociodemographic index.

ASIR and SDI values had nonlinear relationships. The high-income Asia region had the highest ASIR at an SDI of 0.85, while South Asia had the lowest ASIR at an SDI of 0.325. At the regional level, ASIR was higher in the six regions based on the SDIs, including the eastern region. Central and Western Europe, Australasia, Southern Latin America, and high-income North America are regions with ASIR that exceed global levels (Figure 4). The same trend was also observed among the ASMR, ASDR, and SDI values (**Suppl Figures 4 & 5**).

**Figure 5:**
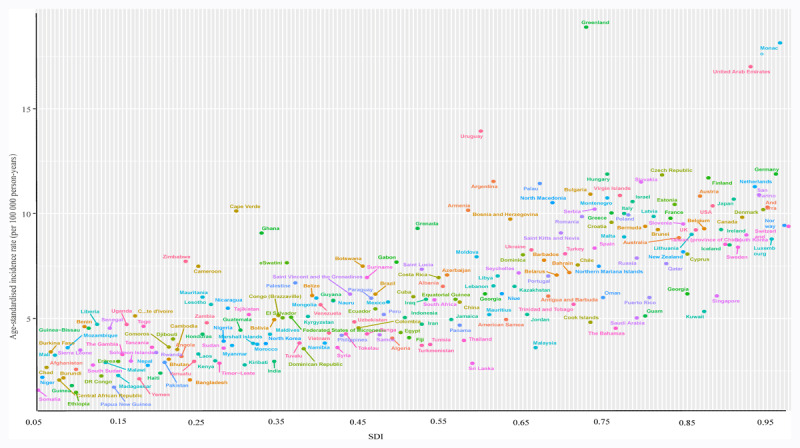
ASIR of pancreatic cancer in 204 countries by SDI, 1990–2019. ASIR, age-standardized incidence rate; SDI, sociodemographic index.

Nationally, a nonlinear correlation between ASIR and SDI values was observed. The highest ASIR was found in Greenland when the SDI value was 0.761, followed by that in Monaco and the United Arab Emirates. Most countries had a higher ASIR than expected. The lowest ASIR was found in Ethiopia, with an SDI of 0.343, whereas Ethiopia, Somalia, Papua New Guinea, Guinea, and numerous other countries recorded a lower ASIR (Figure 5). The same trend was also observed in the ASMR, ASDR, and SDI values (**Suppl Figures 6 & 7**).

### Risk factors of pancreatic cancer

Globally, 531,107 (491,948–566,537) deaths were due to PC, with an ASMR of 6.6 (6.1–7.1) per 100,000 person-years, and an increase was noticed in EAPC (0.77; 0.73–0.81). The proportion of PC deaths in 2019 could be attributed to three risk factors with GBD estimates: smoking, 21.4%; elevated fasting glucose, 9.1%; and elevated body mass index (BMI), 6%. These three risk factors showed different proportions according to sex across the regions. For instance, the maximum proportion attributable to smoking for men was in Eastern Europe (33.2%) and East Asia (31.8%). Meanwhile, the highest proportion attributable to smoking for women was in high-income North America (30.5%) and Central Europe (25.8%). In 2019, the highest proportion of deaths attributable to high fasting glucose levels was observed in Oceania (males, 14.2%; females, 13.1%). Regarding BMI, the highest proportion was observed in Central Europe (7.9%) for men and Eastern Europe (11.6%) for women (**Suppl Figures 8**).

## Discussion

The epidemiology of PC varies widely worldwide owing to different exposure factors. Pancreatic cancer has been ranked as the 11th most common cancer in the world, accounting for 4.5% of all cancer deaths [11]. Early diagnosis is difficult because of its rapid growth, strong invasiveness, and high degree of malignancy. At present, the survival rate is less than 9%, and the total surgical resection rate is 15%, indicating that PC remains a serious public social issue [12,13]. Therefore, we need to further understand the risk factors of PC, which is particularly important for its prevention.

In 2019, the number of incident cases and deaths was 0.20 million and 4.6 million DALYs by GBD. From 1990 to 2019, regardless of region and country, the ASRs of PC for both sexes continuously increased. This study is the first to estimate the number, incidence, ASR, and EAPC of mortality and DALYs for PC worldwide from 1990 to 2019. The increased incidence is bound to increase the medical burden and also affect healthcare quality. These phenomena are widespread in low-and middle-income countries. Thus, better medical resources need to be allocated to these areas to improve healthcare quality.

Our analysis revealed that the high SDI quintiles had the highest incidence and mortality of PC, while the low- or low-middle SDI quintiles had the lowest in 2019. However, the low-middle region showed the most significant increase. This finding indicates a close association between PC and regional development. Early detection and diagnosis have become increasingly difficult because of a lack of medical resources. Low-income levels have delayed the diagnosis and treatment in these areas, resulting in higher mortality and national burden. At the national level, we analyzed the ASMR across 204 countries and territories using the SDI in 2019. We found that Greenland still ranked first for mortality rate at the national level, followed by Monaco and the United Arab Emirates.

A previous study by the Global Cancer Observatory revealed that PC poses a substantial impact in terms of both incidence and death cases [14]. Collectively, these results indicate that these cases have risen and are expected to continue to rise in the future. The number of PC cases was 2.7 times higher in 2019 than those in 1990. Interestingly, we found a higher proportion of male patients than of female patients. This phenomenon is not completely understood, but these results are in line with comparable studies reported [3,15]. Additionally, the incidence and mortality rates in developed countries are significantly higher. It is promising that the ASIR in Southern Latin America (10.5 per 100,000) was increased, and the countries with the lowest ASIR were in Oceania.

HDI is an essential indicator for measuring levels of human development and socioeconomic progress [16]. We performed a nonlinear correlation analysis of the relationship between EAPCs and HDI. The results revealed a positive correlation between EAPCs and HDI when HDI < 0.7. In contrast, for HDI values above 0.7, a negative association was observed between EAPC and HDI. These findings may be explained by the following reasons. First, there are potential differences among populations in different regions, including lifestyle habits, such as the frequency of smoking and the prevalence of metabolic syndrome. Second, individuals with a higher education level and income care more about their health, particularly prevention, early diagnosis, and treatment [17,18].

Smoking has been shown by several prospective studies to increase the risk and reduce the survival of PC [19,20,21]. However, this modifiable risk factor can be controlled by a change in lifestyle habits [22]. A large meta-analysis of 2,517,623 participants revealed that compared with non-smokers, current smokers (56%) and previous smokers (15%) had a higher overall mortality risk [23]. Another meta-analysis of 82 studies revealed that smokers have a 75% higher risk of getting PC than do non-smokers [24]. Globally, we observed that 21.4% of PC deaths in 2019 were from smoking. Among the three most common attributable causes of death, smoking was associated with the highest number of deaths (21.4%) (versus high fasting glucose, 9.1% and high BMI, 6%). At the global level, we found that smoking is prevalent among men, which may be responsible for the higher proportion of death in men (26.1%) than in women (16.1%) among PC patients [25].

Although the association of PC with diabetes is complex, diabetes has been regarded as an independent risk factor of PC [26,27,28]. The current analysis found that high fasting glucose is a risk factor that accounts for 9% of deaths. A cohort study indicated that approximately 85% of PC patients were diagnosed with diabetes [29]. Recently, two meta-analyses found that the risk of pancreatic malignancies in diabetes patients is approximately twice that of patients without diabetes [26,30]. Considering the prevalence of diabetes worldwide, PC incidence and mortality rates will remain high in the future.

With changes in lifestyle and improvements in living standards, obesity has become a worldwide burden, with its incidence continuing to increase [31]. Obesity is an important cause of pain and death from many unnecessary cancers. It is a potent risk factor for PC, and the positive correlation between them has been confirmed [32]. The current study found that 6% of PC deaths in 2019 are related to high BMI; among these, 4.9% in men and 7.2% in women. A randomized study involving a large population of postmenopausal women found that the risk of PC in women with a BMI of ≥25 kg/m^2^ was significantly reduced after a low-fat diet intervention [33]. Obesity becomes a serious risk particularly among women because they are generally more prone to obesity.

## Limitations

The primary limitation of our study is the accuracy and robustness of the results with respect to data quality, especially high-quality data in the GBD. Additionally, our findings analyzed only the three main factors affecting PC. Other factors, such as environmental and genetic effects, were not analyzed. Finally, owing to few relevant data, the study fails to assess the burden of different types of PC in different regions.

## Conclusions

The incidence rate and mortality of PC worldwide have continuously increased from 1990 to 2019 and remain high to date. Therefore, PC remains a serious public health concern worldwide. Our research has updated the epidemiological trends and risk factors of PC. Our findings highlight the urgent need for effective prevention and control strategies for PC. Some examples of the targeted strategies that are required to prevent and treat PC include reducing exposure to modifiable risk factors; improving the early detection and diagnosis of PC through screening high-risk populations; and developing new therapeutic options based on the molecular subtypes and genetic mutations of PC.

## Data Accessibility Statement

The data that support the findings of this study are available from the Global Burden of Disease Study 2019 (GBD 2019) at http://ghdx.healthdata.org/gbd-2019.

## Additional File

The additional file for this article can be found as follows:

10.5334/aogh.4019.s1 Supplementary file. Supplementary Figures s1 to s8 and Table s1.
