# Supplementary material for: Global, Regional, and National Burden of Pancreatic Cancer, 1990–2019: Results from the Global Burden of Disease Study 2019
Source: Ann Glob Health. 2023 May 25;89(1):33. doi: 10.5334/aogh.4019 (PMC10215993; doi:10.5334/aogh.4019)
Supplement: Supplementary file. — Supplementary Figures s1 to s8 and Table s1. [file agh-89-1-4019-s1.pdf]

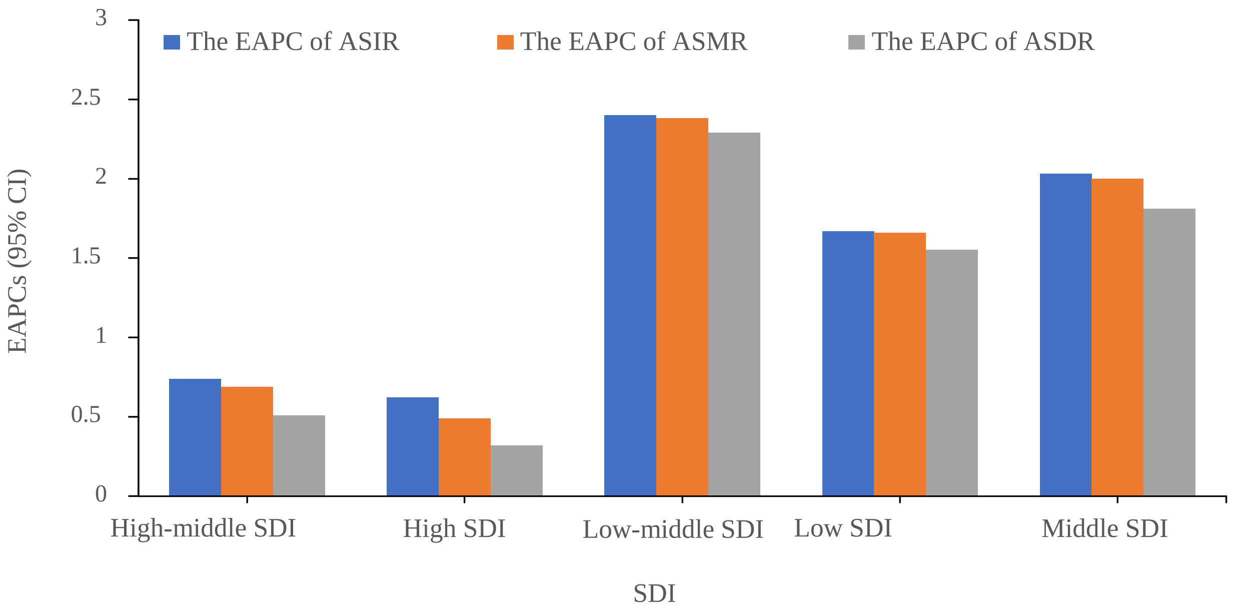

Supplementary Figure 1. The EAPCs of pancreatic cancer ASRs from 1990 to 2019, both sexes by SDI.

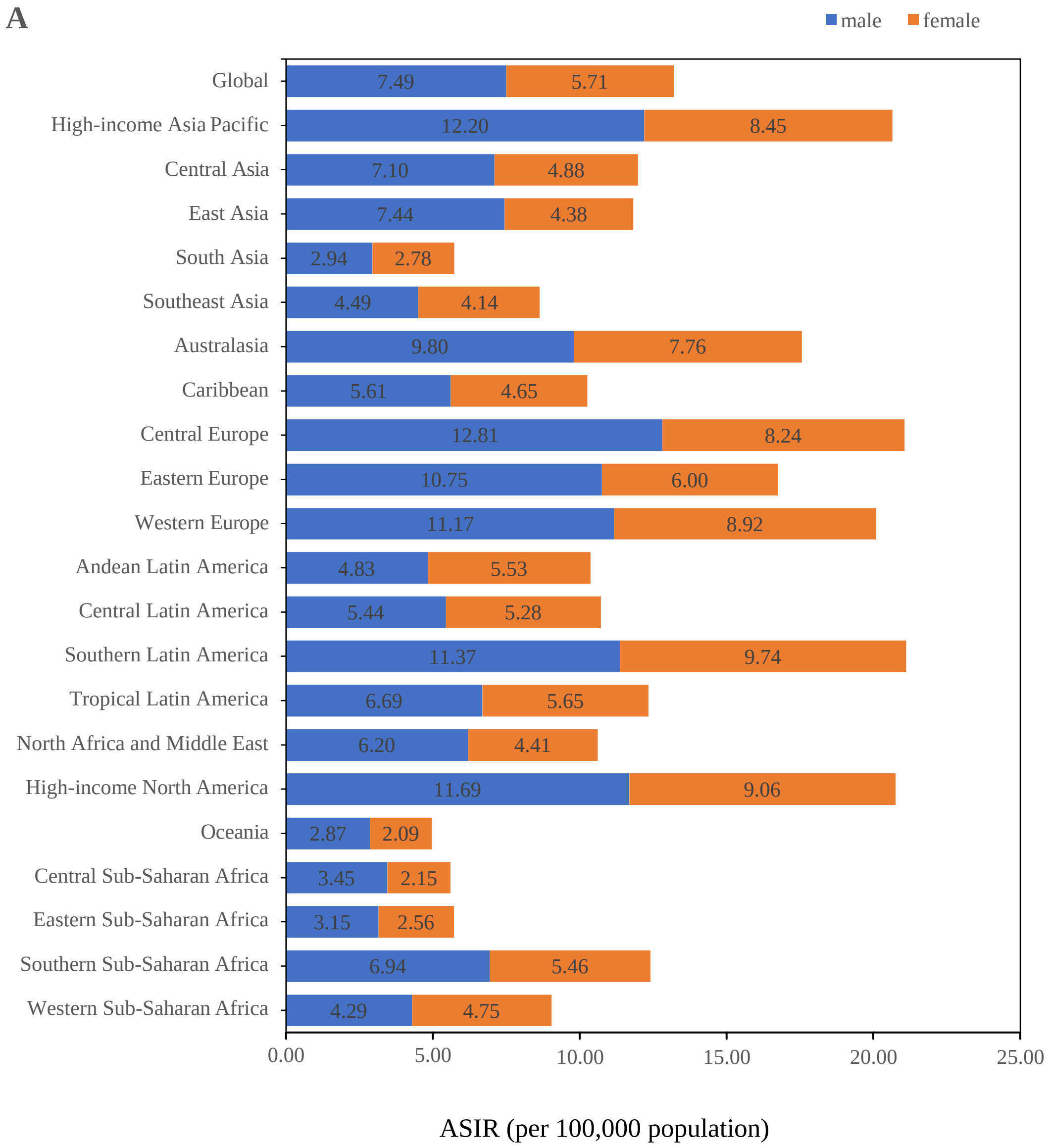

Supplementary Figure 2. The regional burden of pancreatic cancer for both sexes in 2019.

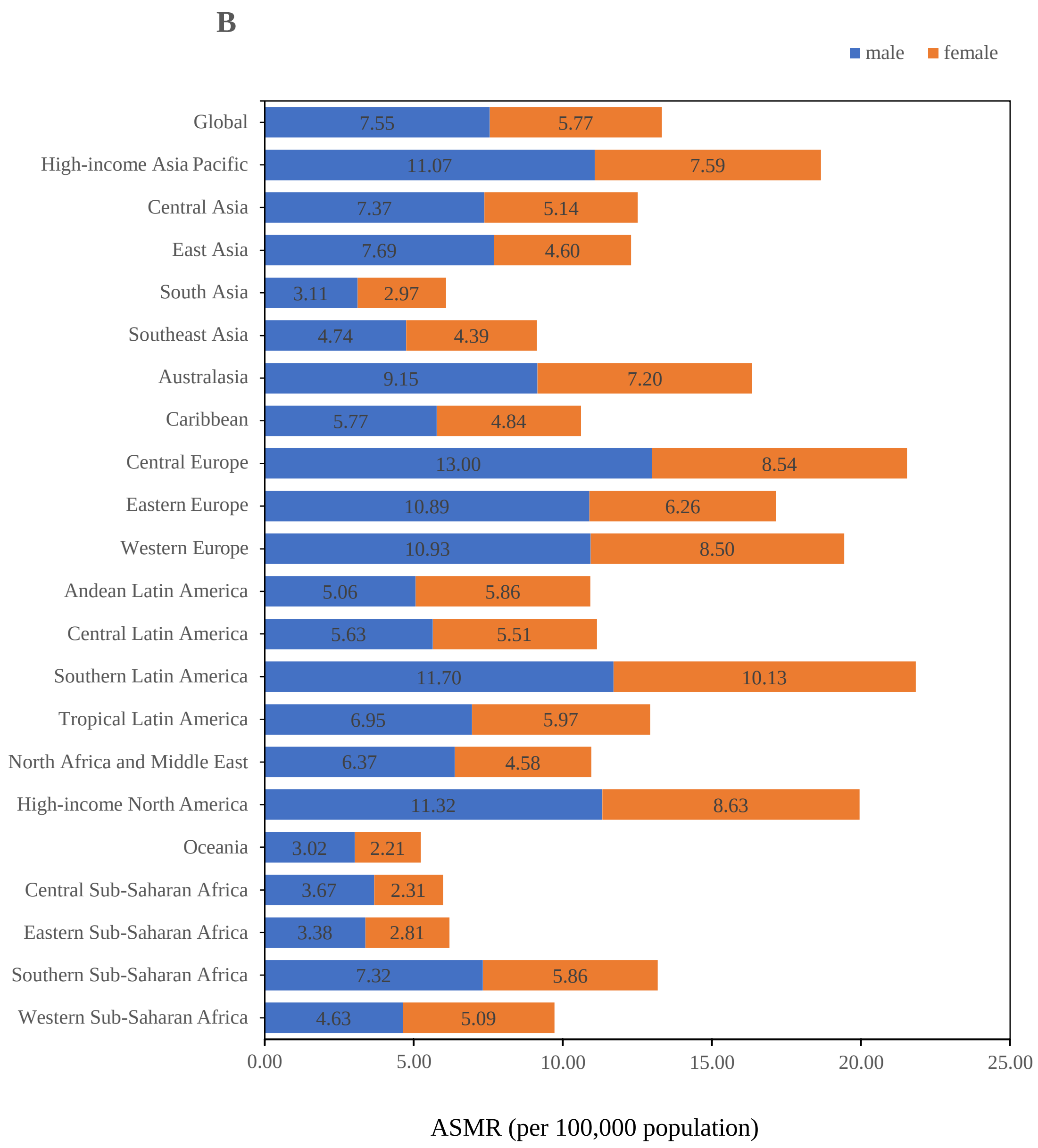

Supplementary Figure 2. The regional burden of pancreatic cancer for both sexes in 2019.

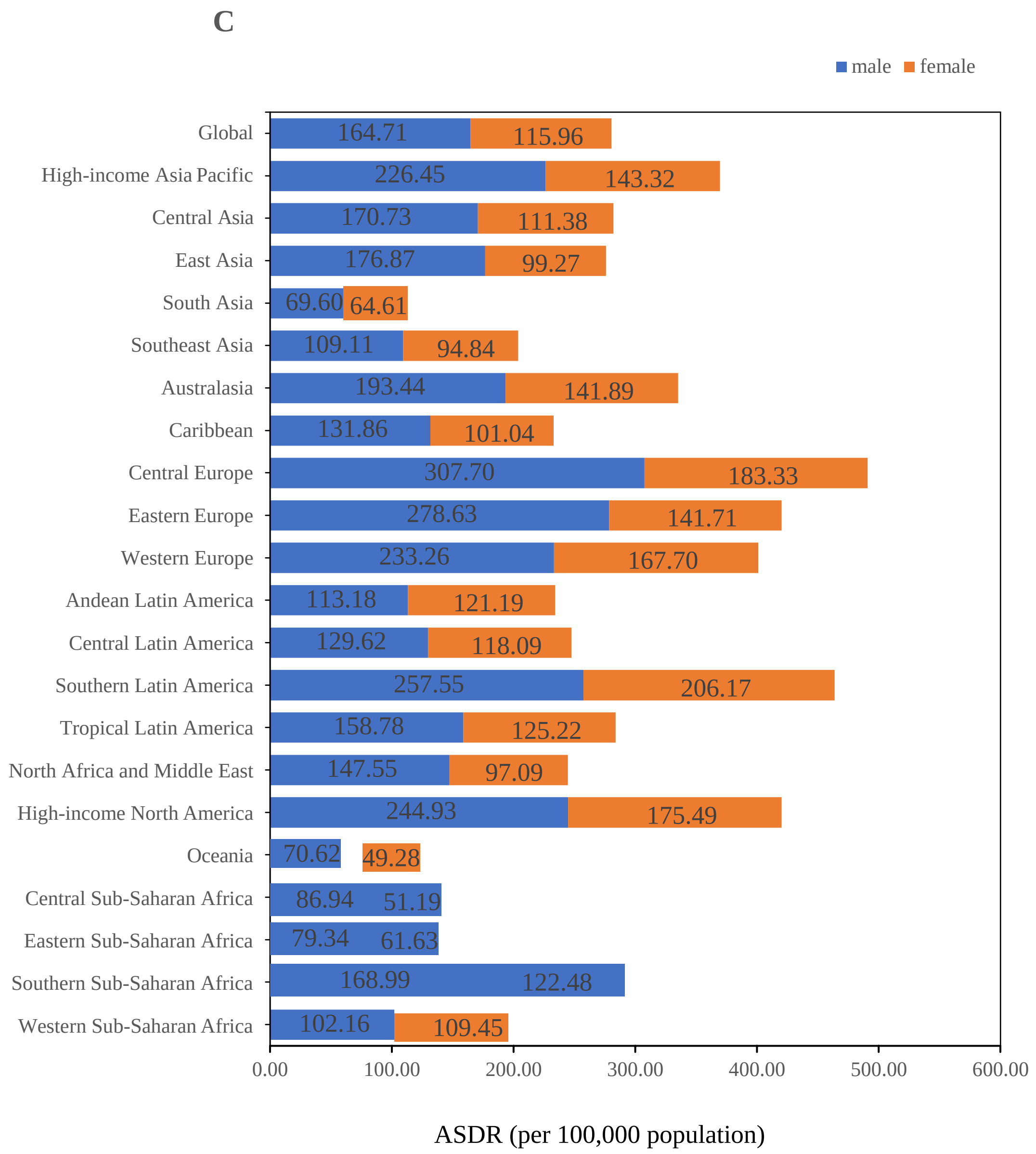

Supplementary Figure 2. The regional burden of pancreatic cancer for both sexes in 2019.

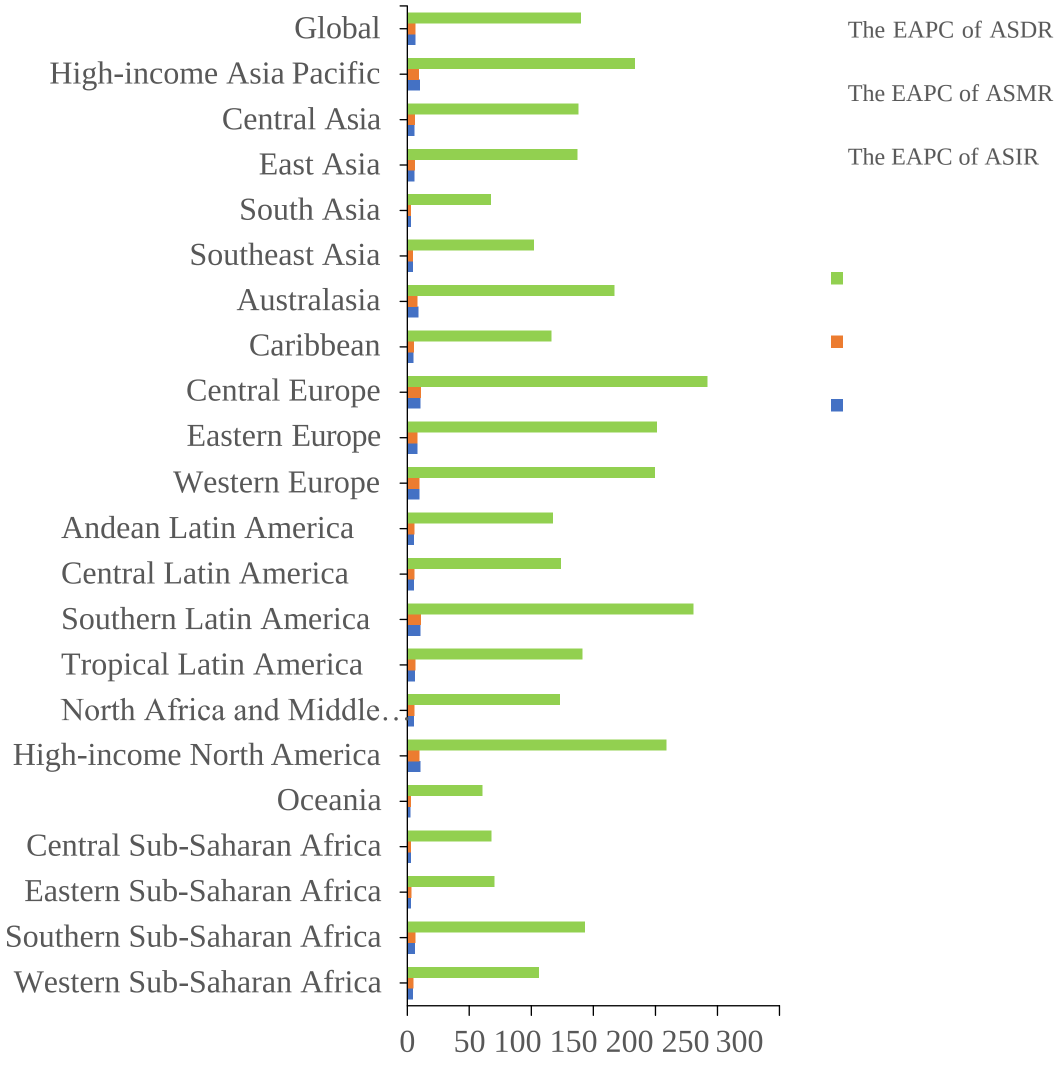

Supplementary Figure 3. The EAPCs of pancreatic cancer at regional level, from 1990 to 2019.

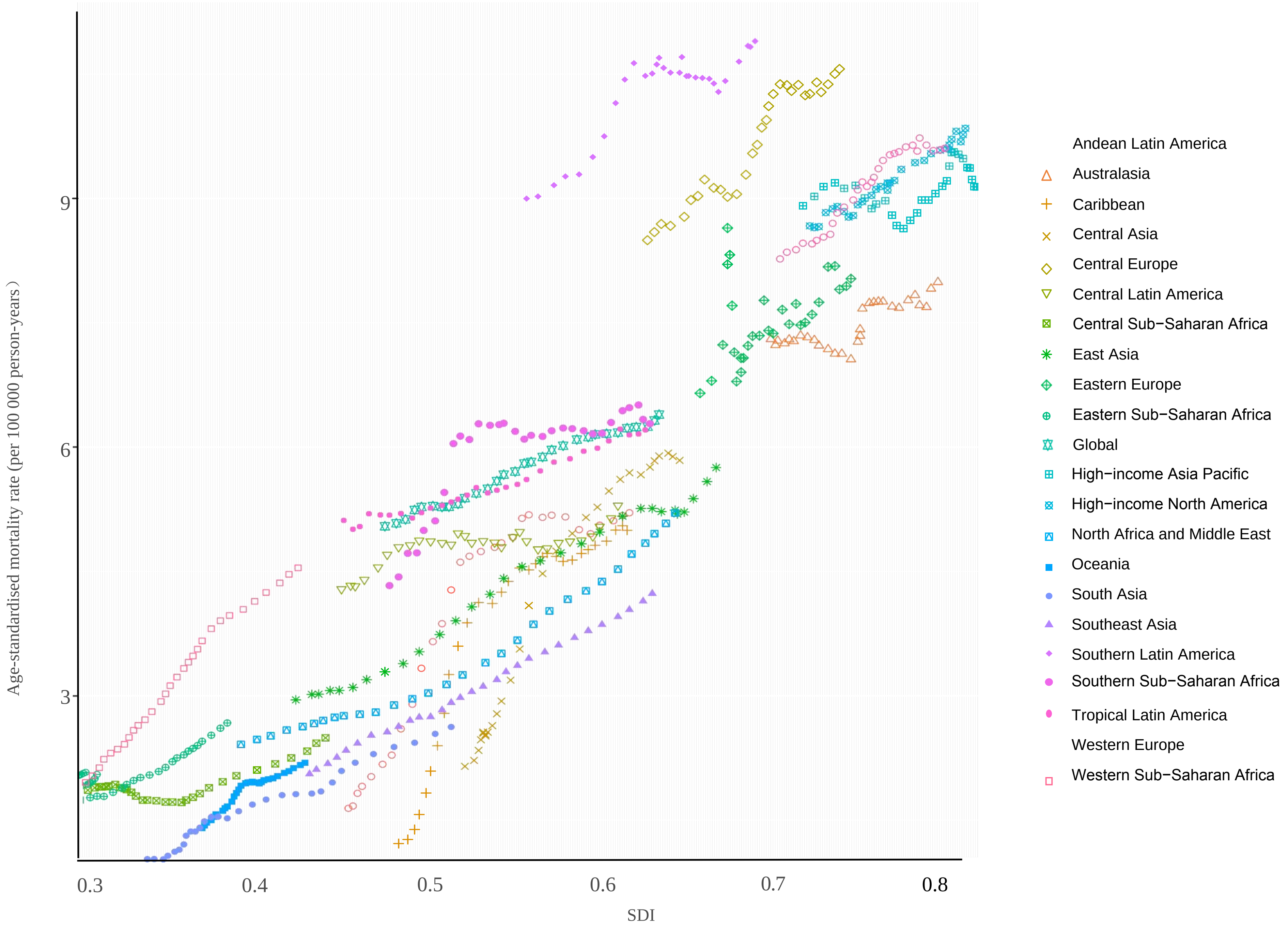

Supplementary Figure 4. ASMR of pancreatic cancer in global and 21 GBD regions by SDI, 1990-2019.

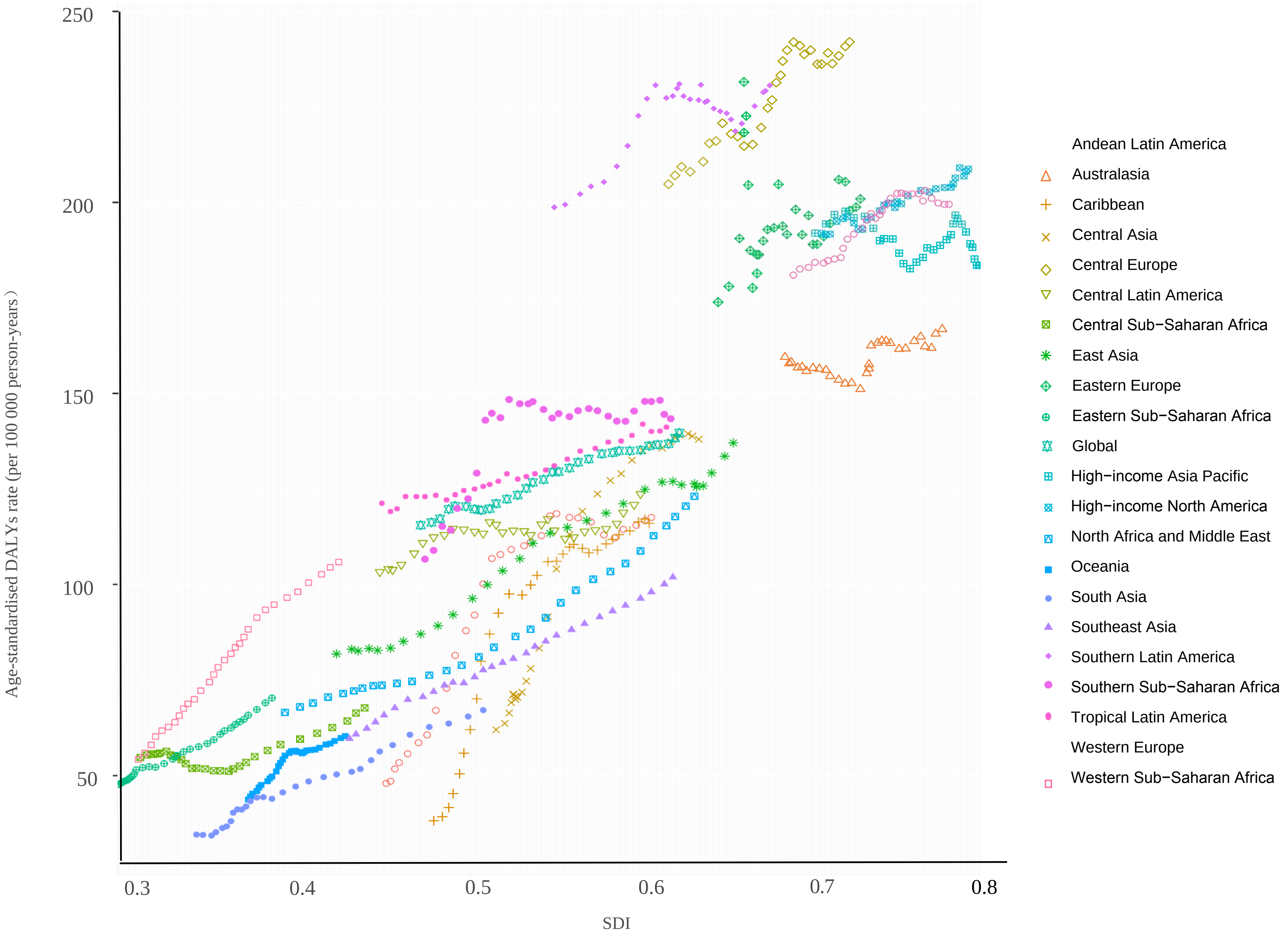

Supplementary Figure 5. ASDR of pancreatic cancer in global and 21 GBD regions by SDI, 1990-2019.

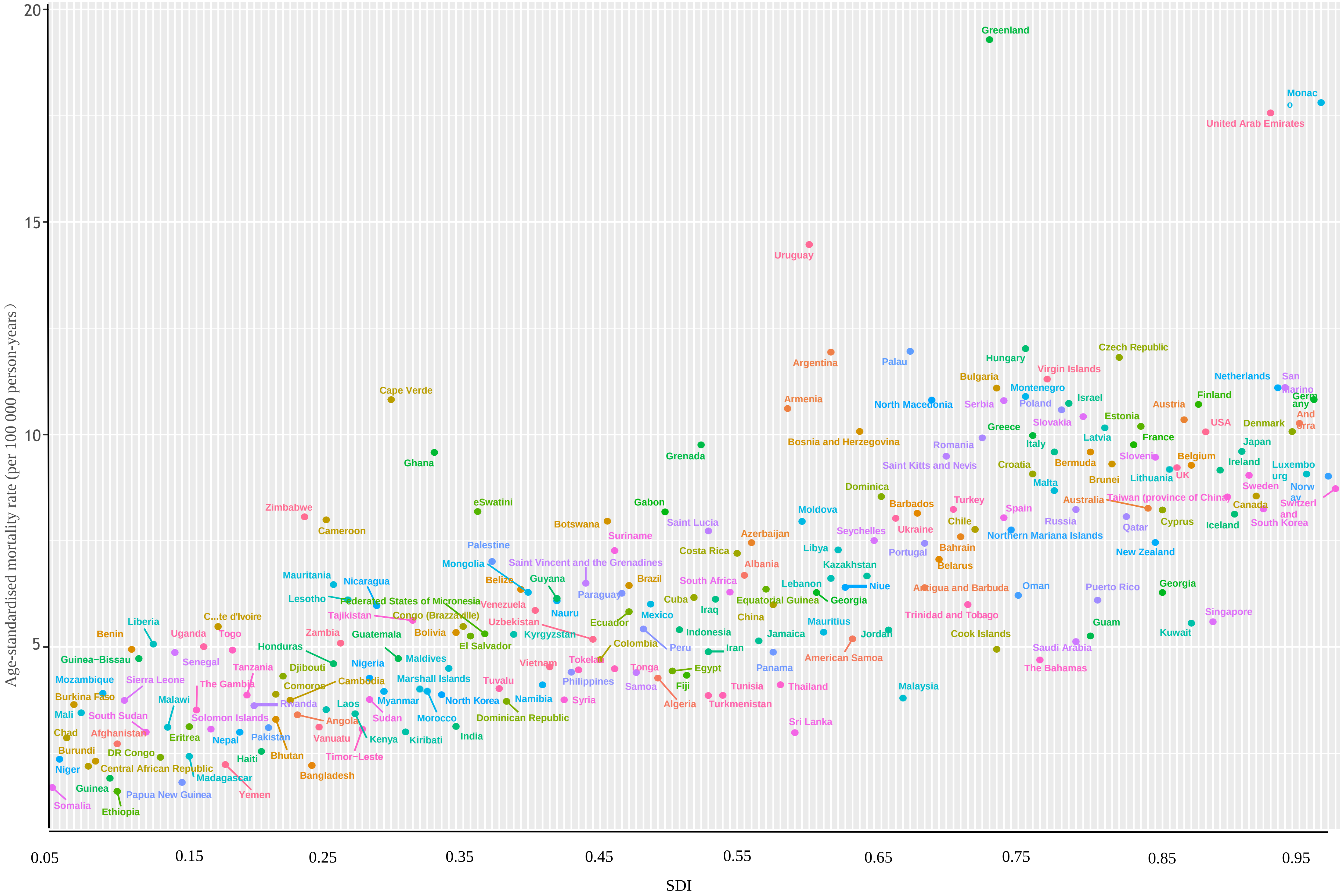

Supplementary **Figure 6. ASMR of pancreatic cancer in 204 countries by SDI, 1990-2019.**

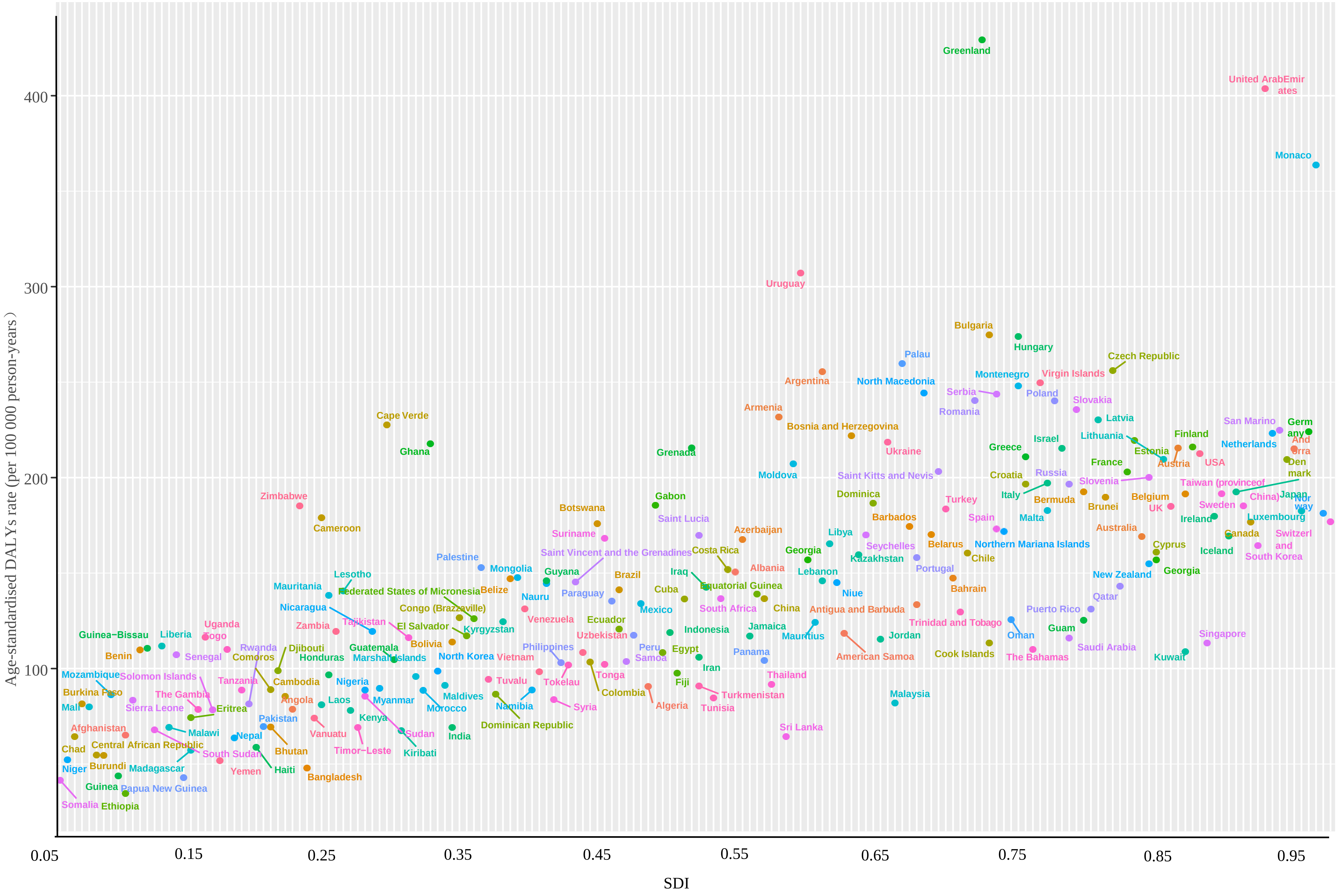

Supplementary **Figure 7. ASDR of pancreatic cancer in 204 countries by SDI, 1990-2019.**

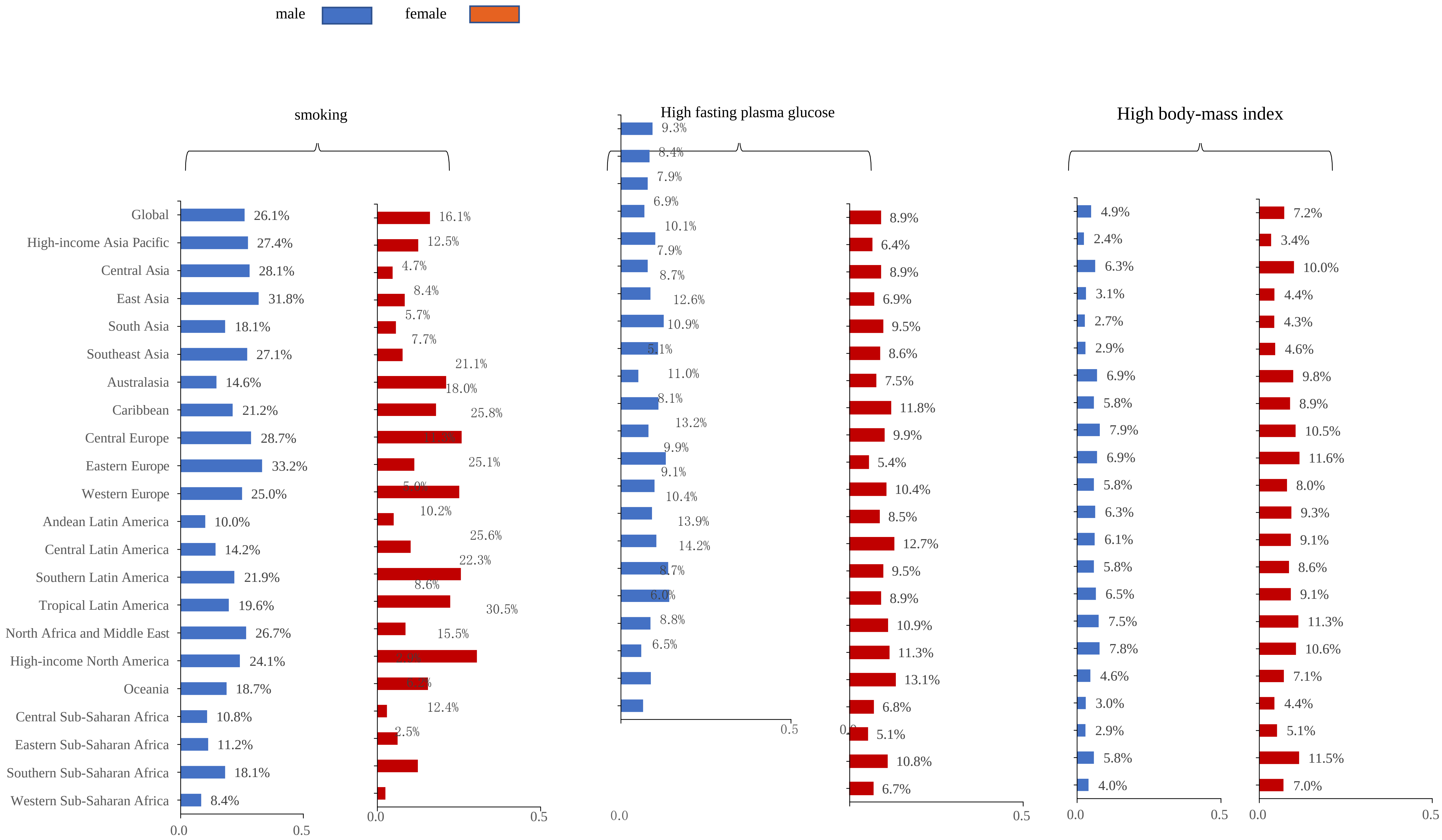

Supplementary Figure 8. The age-standardized deaths attributable to smoking, high fasting plasma glucose, and high body-index.

Table S1. The incident cases, death, and DALYs of Pancreatic Cancer and ASRs per 100,00 population from 1990 and 2019at national level.

location	Cases in 1990	ASIR in 1990	Cases in 2019	ASIR in 2019	EAPC (95% CI)
Afghanistan	134.3 (80-228.9)	1.9 (1.1-3.2)	317.4 (209.6-478.8)	2.6 (1.8-3.7)	1.38 (1.1-1.66)
Albania	75 (69.3-80.7)	3.7 (3.4-3.9)	280.7 (210.6-366.9)	6.5 (4.9-8.6)	2.58 (2.31-2.84)
Algeria	236 (188.5-291.4)	2.1 (1.7-2.5)	1294.1 (1030.2-1587.7)	4.1 (3.3-4.9)	2.32 (2.28-2.37)
American Samoa	0.7 (0.6-0.8)	3.1 (2.6-3.6)	2.3 (1.9-2.8)	4.9 (4.1-5.9)	1.67 (1.25-2.1)
Andorra	4.9 (3.7-6.8)	9.2 (7-12.5)	14.6 (11.1-18.7)	10.3 (7.8-13.2)	0.34 (0.31-0.37)
Angola	78.8 (56.9-107.2)	2 (1.5-2.7)	342.8 (268.5-444.3)	3.2 (2.6-4)	1.38 (1.19-1.56)
Antigua and Barbuda	0.7 (0.7-0.8)	1.4 (1.2-1.5)	6 (5-7)	6.1 (5.1-7.1)	4.9 (4-5.81)
Argentina	2899.1 (2625.9-3182.8)	9 (8.2-9.9)	6254.4 (4896.9-7819.1)	11.5 (9-14.4)	0.62 (0.42-0.83)
Armenia	176.3 (150-205.7)	6.6 (5.6-7.7)	421.9 (350.9-502.9)	10.2 (8.4-12.1)	1.58 (1.31-1.85)
Australia	1526.6 (1456.2-1585.6)	7.8 (7.4-8.1)	3779.2 (2928.7-4729.8)	8.9 (6.9-11.1)	0.45 (0.38-0.52)
Austria	1267.5 (1206.6-1319.8)	10.6 (10.2-11.1)	1963.9 (1603.8-2384.2)	10.8 (8.8-13.2)	0.03 (-0.05-0.11)
Azerbaijan	170.3 (149.3-200.2)	3.4 (3-4)	627.5 (530.3-761.5)	7.1 (6-8.6)	2.98 (2.7-3.26)
Bahamas	2.7 (2.5-3)	1.8 (1.6-2)	17.5 (14.1-21.6)	4.5 (3.7-5.6)	3.44 (2.72-4.17)
Bahrain	10 (8.5-11.8)	6.3 (5.4-7.4)	59.3 (46-75)	7.2 (5.7-8.8)	0.05 (-0.26-0.37)
Bangladesh	589.7 (372.8-823.6)	1.3 (0.8-1.8)	2616.2 (1651.7-3878.6)	2.1 (1.3-3.1)	1.62 (1.52-1.73)
Barbados	4.2 (3.8-4.6)	1.4 (1.3-1.6)	38.4 (31.2-45.6)	7.8 (6.3-9.3)	6.05 (4.78-7.34)
Belarus	637.7 (594.4-684.7)	4.9 (4.6-5.2)	1118.4 (874.3-1444.6)	7.1 (5.5-9.2)	1.12 (0.97-1.28)
Belgium	1391.7 (1309.9-1466.5)	9 (8.5-9.4)	2154.1 (1706.6-2691.1)	9.3 (7.3-11.7)	0.27 (-0.01-0.56)
Belize	1.4 (1.3-1.6)	1.6 (1.4-1.7)	16.6 (13.9-19.4)	6.1 (5.1-7.1)	4.51 (3.26-5.78)
Benin	42 (35.7-49.4)	2.2 (1.8-2.5)	213.1 (164.3-275.7)	4.6 (3.6-5.9)	2.76 (2.52-3)
Bermuda	1.7 (1.6-1.9)	2.8 (2.6-3.1)	12.5 (10.3-15.3)	9.4 (7.8-11.5)	3.86 (2.68-5.05)
Bhutan	3.1 (1.6-4.9)	1.4 (0.7-2.1)	16.4 (9.3-24.9)	3.1 (1.7-4.6)	3.02 (2.94-3.1)
Bolivia (Plurinational State of)	73.7 (53.6-93.7)	2.4 (1.7-3)	421.8 (288.7-568.5)	4.9 (3.4-6.7)	2.4 (2.22-2.59)
Bosnia and Herzegovina	298.6 (280.4-318.4)	7.5 (7-8)	584.3 (464.9-726.5)	9.7 (7.8-12.1)	0.83 (0.74-0.92)
Botswana	19.4 (15.1-24.7)	3.6 (2.8-4.5)	95.9 (68.1-132.7)	7.5 (5.5-10)	2.24 (1.94-2.53)
Brazil	4430.8 (4258.5-4576.6)	5.2 (4.9-5.4)	14429.6 (13346.8-15276.8)	6.2 (5.7-6.5)	0.68 (0.62-0.74)
Brunei Darussalam	5.6 (4.7-6.6)	6.4 (5.5-7.5)	23.9 (20.5-27.4)	9.2 (8-10.5)	1.62 (1.43-1.8)
Bulgaria	842.5 (793.2-892.9)	6.7 (6.3-7.1)	1525.3 (1211.1-1907.3)	10.9 (8.6-13.7)	3.03 (2.54-3.52)
Burkina Faso	72.7 (56.2-93.7)	1.7 (1.3-2.2)	295 (221.8-376)	3.4 (2.6-4.3)	2.39 (2.32-2.46)
Burundi	52.4 (39.5-66.3)	2.2 (1.7-2.8)	96.8 (70-136.1)	2.2 (1.6-3)	-0.33 (-0.43--0.24)
Cabo Verde	2.7 (2.4-3.1)	1.2 (1.1-1.3)	41.7 (32.8-50.7)	10.1 (7.9-12.3)	6.61 (5.38-7.85)
Cambodia	93.7 (69.6-122.2)	2.1 (1.6-2.7)	406.5 (322.8-490.9)	3.5 (2.8-4.2)	1.91 (1.79-2.02)
Cameroon	156.2 (121.4-192.3)	3.6 (2.8-4.4)	846.4 (585-1172.9)	7.5 (5.3-10.2)	2.44 (2.33-2.55)
Canada	2952.7 (2800.8-3067.7)	9.1 (8.6-9.5)	6909.6 (5399.7-8642.6)	9.8 (7.6-12.3)	0.27 (0.21-0.34)

Central African Republic	23.9 (16.1-33)	2 (1.5-2.7)	45.1 (29.3-63)	2.1 (1.5-2.8)	0.05 (0-0.1)
Chad	36.5 (28.5-45.9)	1.3 (1-1.6)	142.4 (112.8-179.5)	2.7 (2.1-3.3)	2.53 (2.37-2.68)
Chile	650.9 (610.1-692.9)	6.7 (6.3-7.2)	1822.2 (1416.9-2303.6)	7.6 (5.9-9.6)	0.53 (0.46-0.6)
China	26773.1 (23130.3-30261.6)	3.2 (2.8-3.6)	114964.2 (98047.5-133708.1)	5.8 (4.9-6.7)	2.32 (2.13-2.51)
Colombia	888.4 (844-931.7)	5.2 (4.9-5.5)	2406.5 (1855.5-3050.3)	4.5 (3.5-5.8)	-0.68 (-0.91--0.45)
Comoros	5.9 (3.4-7.6)	2.7 (1.6-3.4)	17.3 (13.2-22)	3.6 (2.8-4.6)	0.79 (0.65-0.93)
Congo	39 (26.1-53.2)	3.7 (2.6-4.9)	129.9 (82.8-190.1)	5.1 (3.4-7.3)	0.95 (0.71-1.19)
Cook Islands	0.5 (0.4-0.5)	3.6 (3-4.4)	1.2 (1-1.4)	4.8 (4.1-5.7)	0.68 (0.52-0.84)
Costa Rica	52.5 (48-57.2)	3.1 (2.8-3.4)	354.4 (275.2-445.9)	6.9 (5.4-8.8)	2.49 (1.96-3.03)
Croatia	546.5 (509.7-583.6)	8.6 (8-9.2)	851.4 (680.9-1067.3)	9.6 (7.6-12)	0.74 (0.57-0.9)
Cuba	172.4 (160.4-184.4)	1.7 (1.6-1.8)	1151.2 (938.9-1409.9)	6 (4.9-7.4)	4.36 (3.32-5.41)
Cyprus	31.6 (28.2-35.4)	4.1 (3.6-4.5)	155.1 (134.3-178.2)	8.1 (7-9.2)	2.99 (2.61-3.36)
Czechia	1604.7 (1539.6-1668)	11.6 (11.2-12.1)	2507.6 (2032.9-3027.5)	11.8 (9.6-14.3)	0.28 (0.11-0.46)
Côte d'Ivoire	102.2 (81.2-127.2)	2.6 (2.1-3.2)	509 (386.6-650.9)	5.1 (4-6.4)	1.95 (1.7-2.19)
Democratic People's Republic of Korea	478.4 (361.8-629.4)	2.9 (2.2-3.7)	1229.5 (916.5-1575)	3.8 (2.9-4.8)	1.05 (1-1.1)
Democratic Republic of the Congo	310.4 (244-397.1)	2 (1.6-2.6)	780.5 (590.2-1013.7)	2.3 (1.7-2.9)	-0.01 (-0.36-0.35)
Denmark	600.2 (567.1-629.5)	7.4 (7-7.7)	1185.7 (919.5-1506.5)	10.2 (7.9-12.9)	1.05 (0.64-1.47)
Djibouti	3.3 (2.4-4.5)	2.4 (1.8-3.2)	23.1 (15.8-33.1)	4 (2.9-5.5)	1.78 (1.69-1.87)
Dominica	1.3 (1.1-1.4)	1.8 (1.6-2)	7.3 (5.8-8.9)	8 (6.4-9.8)	5.42 (4.48-6.37)
Dominican Republic	50.8 (44.4-58)	1.4 (1.2-1.6)	327.5 (222.9-447.2)	3.6 (2.4-4.8)	4.13 (3.64-4.63)
Ecuador	93 (87.1-99.2)	1.8 (1.7-1.9)	799.9 (634.1-1009)	5.5 (4.4-6.9)	3.54 (2.8-4.28)
Egypt	590.6 (532.8-643.1)	2 (1.8-2.2)	2800.7 (1917.8-3942.1)	4.3 (3-6)	2.47 (2.29-2.65)
El Salvador	48.8 (45.3-52.7)	1.7 (1.5-1.8)	298.4 (224.3-383.5)	5 (3.8-6.5)	3.93 (3.32-4.55)
Equatorial Guinea	3.6 (2.3-5.4)	1.8 (1.2-2.7)	26.6 (15.6-41.4)	5.9 (3.6-8.8)	4.89 (4.64-5.13)
Eritrea	17.4 (12.5-23.2)	1.7 (1.2-2.3)	77 (51.3-108.2)	3 (2-4.1)	1.59 (1.31-1.86)
Estonia	175.5 (163.9-187.2)	8.6 (8-9.1)	279 (218.7-348.5)	10.4 (8.1-13.2)	0.78 (0.54-1.03)
Eswatini	12.6 (9.4-16.6)	4.5 (3.4-5.9)	42.3 (28.3-59.5)	7.7 (5.2-10.6)	1.39 (0.72-2.06)
Ethiopia	228 (133.2-356.8)	1.2 (0.7-1.9)	573.8 (384.3-819.9)	1.5 (1-2.1)	0.75 (0.52-0.98)
Fiji	8.9 (7.3-10.8)	2.6 (2.1-3.1)	29 (22.6-36.6)	4.1 (3.2-5.1)	1.51 (1.44-1.58)
Finland	807.3 (767.3-843.1)	11.2 (10.6-11.7)	1486.2 (1171.6-1873.6)	11.7 (9.2-14.8)	0.4 (0.29-0.51)
France	6381.1 (6051.7-6646.6)	7.7 (7.3-8)	13628.2 (10633.1-17159.7)	9.8 (7.6-12.4)	1.02 (0.9-1.14)
Gabon	21.3 (16.1-33.4)	3.9 (3-5.9)	77.7 (52-109.4)	7.7 (5.3-10.6)	2.17 (2.02-2.33)
Gambia	5.1 (3.8-6.5)	1.5 (1.2-1.9)	30.2 (20.6-41.7)	3.3 (2.2-4.5)	2.57 (2.38-2.76)
Georgia	237 (193.7-306.2)	3.8 (3.1-4.9)	359.8 (295.7-427.4)	6.2 (5.1-7.4)	2.6 (2.02-3.18)
Germany	10930.8 (10394.9-11389.6)	8.6 (8.2-8.9)	22969.5 (17938.1-29284.3)	11.9 (9.3-15.3)	1.19 (0.95-1.43)

Ghana	264.3 (216.2-322.2)	4.4 (3.6-5.2)	1407.5 (1066.1-1845.6)	9.1 (6.9-12)	2.33 (2.2-2.46)
Greece	1396.8 (1321.1-1468.1)	9.1 (8.6-9.6)	2404 (1896-3035.6)	10 (7.9-12.7)	0.09 (0-0.18)
Greenland	5.1 (4.5-5.8)	15.4 (13.7-17.3)	12.9 (10.4-15.2)	18.9 (15.5-22.3)	0.4 (0.1-0.69)
Grenada	1.2 (1.1-1.4)	1.7 (1.5-1.9)	10.2 (8.9-11.6)	9.3 (8.2-10.5)	6.27 (5.18-7.37)
Guam	2.9 (2.4-3.3)	4 (3.4-4.7)	9.6 (8-11.5)	5.1 (4.2-6.1)	1.12 (0.9-1.35)
Guatemala	52.7 (45.4-61.2)	1.5 (1.3-1.7)	482.3 (373.7-605.8)	4.4 (3.5-5.6)	3.95 (3.12-4.78)
Guinea	36.5 (29.5-44.1)	1.1 (0.9-1.4)	95.8 (72.1-121)	1.8 (1.4-2.3)	1.62 (1.52-1.71)
Guinea-Bissau	10.5 (7-14.2)	2.6 (1.8-3.5)	31.6 (20.9-43.6)	4.5 (3.1-6.1)	1.99 (1.86-2.12)
Guyana	6.3 (5.4-7.3)	1.7 (1.5-2)	36.3 (27.5-46.3)	5.9 (4.5-7.4)	4.14 (3.29-4.99)
Haiti	38 (28.9-47.5)	1.2 (0.9-1.5)	161.9 (109.3-227.9)	2.4 (1.7-3.3)	2.71 (2.52-2.89)
Honduras	38.8 (31-45.6)	1.9 (1.6-2.3)	243.9 (146.2-353.8)	4.2 (2.6-6.1)	2.85 (2.71-2.99)
Hungary	1450.8 (1388.7-1519.3)	9.9 (9.5-10.4)	2271.4 (1868.7-2723.7)	11.9 (9.8-14.3)	0.64 (0.49-0.78)
Iceland	25.2 (23-27.6)	8.8 (8-9.6)	47.5 (40.6-55.5)	8.5 (7.3-9.9)	-0.14 (-0.24--0.04)
India	6081.4 (4968.9-7205.2)	1.4 (1.2-1.7)	32481.9 (28159.9-37342.9)	3 (2.5-3.4)	2.52 (2.38-2.65)
Indonesia	2150.8 (1782.9-2510.6)	2.2 (1.9-2.6)	10322.5 (6797.3-14365.5)	5 (3.3-7)	2.89 (2.83-2.94)
Iran (Islamic Republic of)	532.4 (433.4-645.6)	2.1 (1.7-2.6)	3319.2 (3082.3-3604.6)	4.7 (4.4-5.1)	2.86 (2.7-3.03)
Iraq	230.1 (159.4-310.9)	3 (2.1-4.1)	1332.8 (1002.9-1667.6)	5.9 (4.5-7.3)	2.61 (2.25-2.97)
Ireland	391.4 (369.2-413.6)	9.5 (9-10.1)	700.5 (542.2-884.9)	9.2 (7.2-11.7)	-0.04 (-0.13-0.05)
Israel	441.1 (413.9-467.3)	9.1 (8.5-9.6)	1238.8 (968.2-1571.2)	10.6 (8.2-13.4)	0.33 (0.23-0.43)
Italy	7946.6 (7681-8133.2)	8.9 (8.6-9.1)	14685.8 (12030.3-17333.8)	10 (8.3-11.8)	0.41 (0.29-0.53)
Jamaica	23.8 (21.7-26)	1.3 (1.2-1.5)	147.3 (115-184.1)	4.9 (3.8-6.2)	4.35 (3.43-5.27)
Japan	16442.6 (15599.8-16888.3)	9.8 (9.2-10)	40981.4 (33124.7-47874.9)	10.7 (8.8-12.5)	0.54 (0.43-0.66)
Jordan	32.2 (26.8-38.5)	2.6 (2.1-3.1)	316.7 (263.4-375.8)	5.2 (4.3-6.2)	2.85 (2.68-3.02)
Kazakhstan	147.3 (132-168.1)	1.2 (1.1-1.4)	1130.5 (962.8-1313.5)	6.5 (5.6-7.6)	7.66 (6.53-8.81)
Kenya	111.9 (72.8-163.4)	1.4 (0.9-2)	632.6 (488.3-830.8)	3 (2.3-3.9)	2.87 (2.73-3)
Kiribati	0.8 (0.6-0.9)	2.2 (1.8-2.6)	1.7 (1.3-2.3)	2.8 (2.1-3.6)	0.31 (0-0.63)
Kuwait	21.6 (18.9-24.3)	3.9 (3.4-4.4)	121.3 (97.8-148.1)	5.3 (4.3-6.5)	1.71 (1.32-2.1)
Kyrgyzstan	112.6 (98.6-128.2)	3.7 (3.2-4.2)	229.9 (197.4-264.6)	5.1 (4.4-5.8)	1.16 (0.96-1.35)
Lao People's Democratic Republic	47.7 (33.5-67.5)	2.3 (1.6-3.1)	140.1 (107.8-180.3)	3.3 (2.6-4.1)	1.26 (1.17-1.35)
Latvia	281.3 (262-302.1)	7.8 (7.3-8.4)	391.2 (323.2-477.5)	9.9 (8.1-12.1)	0.52 (0.23-0.81)
Lebanon	70 (57.4-84.6)	3.2 (2.6-3.8)	339.8 (264-415.8)	6.6 (5.1-8)	2.96 (2.75-3.17)
Lesotho	24.4 (18-32.5)	2.6 (1.9-3.4)	68.4 (47.1-93.3)	5.7 (4-7.6)	2.95 (2.79-3.12)
Liberia	27.3 (21.8-33.9)	2.5 (2-3.1)	90.5 (64.1-121)	4.7 (3.4-6.2)	2.92 (2.52-3.33)
Libya	69.1 (43.6-95.9)	3.8 (2.4-5.3)	348.2 (264.5-449.8)	7 (5.4-9)	2.27 (2.12-2.43)
Lithuania	334.1 (314.7-353)	7.4 (7-7.8)	513.2 (417.7-630)	9 (7.3-11.1)	0.57 (0.33-0.81)
Luxembourg	53 (49.3-56.8)	9.7 (9-10.4)	89.3 (73.1-108.1)	8.8 (7.2-10.6)	-0.22 (-0.44-0.01)

Madagascar	95.9 (75.3-113.1)	1.9 (1.5-2.2)	242.8 (170.4-329.4)	2.3 (1.6-3)	0.55 (0.45-0.65)
Malawi	70.8 (58.9-84)	1.9 (1.6-2.2)	199.4 (153.4-255)	2.9 (2.2-3.6)	1.43 (1.31-1.54)
Malaysia	161.9 (138.6-188.5)	1.8 (1.6-2.2)	927.7 (717.5-1170.3)	3.6 (2.8-4.5)	2.51 (2.13-2.88)
Maldives	2.4 (1.6-3.4)	2.8 (2-3.9)	12 (9.9-14.4)	4.2 (3.5-5.1)	1.1 (0.92-1.28)
Mali	85.6 (72.7-100.3)	2.1 (1.8-2.4)	271.4 (202.6-357.9)	3.2 (2.5-4.2)	1.53 (1.49-1.58)
Malta	34.6 (31.2-37.7)	8.1 (7.3-8.8)	82.9 (68.1-99.2)	8.9 (7.3-10.6)	0.51 (0.37-0.65)
Marshall Islands	0.4 (0.3-0.4)	2.3 (1.9-2.7)	1.3 (0.9-1.7)	3.8 (2.8-5)	1.74 (1.65-1.84)
Mauritania	33.4 (24.5-43.3)	3.4 (2.5-4.3)	119.2 (85.4-157.4)	6 (4.4-7.8)	2.03 (1.98-2.09)
Mauritius	30 (27.9-32.3)	4.1 (3.8-4.4)	90.1 (71.8-110.3)	5.2 (4.2-6.3)	0.77 (0.6-0.95)
Mexico	2235.5 (2155.6-2298.6)	5.4 (5.2-5.6)	6674.2 (5744.3-7682.7)	5.8 (5-6.6)	-0.11 (-0.23-0.01)
Micronesia (Federated States of)	1.4 (1.1-1.8)	3.1 (2.5-3.8)	3.5 (2.3-4.8)	5.1 (3.5-6.8)	1.55 (1.31-1.79)
Monaco	8.9 (7-10.7)	12.4 (9.7-15)	17.7 (14.1-21.1)	18.1 (14.4-21.7)	1.54 (1.28-1.8)
Mongolia	31.4 (25.4-37.9)	3.1 (2.5-3.7)	138.3 (105.8-178.6)	6 (4.7-7.5)	2.28 (2.11-2.45)
Montenegro	44.9 (39-52.1)	7.2 (6.2-8.4)	106.4 (89.4-126.4)	10.7 (9-12.7)	1.69 (1.6-1.78)
Morocco	251.3 (202.2-305.9)	1.9 (1.5-2.3)	1145.1 (830.2-1501.1)	3.8 (2.7-4.9)	2.36 (2.15-2.57)
Mozambique	98.4 (81.2-116)	1.8 (1.5-2.1)	367.5 (253.6-527.8)	3.6 (2.5-5.1)	2.88 (2.73-3.02)
Myanmar	531 (395.1-709.3)	2.3 (1.7-3)	1665.9 (1299.4-2161.8)	3.7 (2.9-4.8)	1.84 (1.74-1.94)
Namibia	12.2 (9.8-14.8)	1.7 (1.4-2.1)	51.1 (39.3-66.5)	3.8 (3-4.9)	2.83 (2.7-2.95)
Nauru	0.2 (0.1-0.2)	4.1 (3.1-5.5)	0.2 (0.2-0.3)	5.8 (4-7.9)	0.74 (0.54-0.94)
Nepal	92.6 (52.2-138)	1 (0.6-1.6)	583.5 (350.3-850.7)	2.8 (1.6-4.1)	3.7 (3.41-3.99)
Netherlands	1738.2 (1651.5-1811.7)	8.7 (8.2-9)	3922.1 (3081-4872.6)	11.3 (8.8-14.1)	1.22 (1.06-1.39)
New Zealand	296.7 (277.9-315.7)	7.5 (7-8)	647.6 (537-771.1)	8.2 (6.8-9.7)	0.4 (0.27-0.54)
Nicaragua	33.7 (28-40.6)	2.3 (1.9-2.8)	226.2 (185.8-269.7)	5.5 (4.5-6.5)	2.97 (2.58-3.36)
Niger	37.3 (26.4-48.2)	1.4 (1-1.8)	160.1 (106.3-227.7)	2.2 (1.5-3.1)	1.62 (1.48-1.77)
Nigeria	837.3 (596.5-1118.1)	2 (1.5-2.7)	3071.5 (2338.7-3855.7)	3.9 (3-4.8)	2.39 (2.29-2.48)
Niue	0.1 (0.1-0.1)	3.7 (2.9-4.5)	0.1 (0.1-0.2)	6.2 (4.7-7.9)	1.72 (1.52-1.91)
North Macedonia	122.4 (112.5-133.5)	6.5 (6-7.1)	337.5 (266.6-424.3)	10.5 (8.4-13.1)	1.71 (1.58-1.84)
Northern Mariana Islands	0.6 (0.5-0.7)	3.4 (2.8-4.2)	3.7 (3.1-4.4)	7.5 (6.4-8.7)	3.42 (2.76-4.08)
Norway	614.8 (581.8-640.2)	8.9 (8.4-9.2)	922.4 (784.1-1071.4)	9.4 (8-11)	0.43 (0.27-0.58)
Oman	12.8 (8.3-18.5)	2.1 (1.4-3)	88.4 (73.8-108.8)	6 (5.2-6.9)	4.02 (3.68-4.35)
Pakistan	815.1 (668.3-958)	1.5 (1.2-1.7)	3032.8 (2376.3-3954.9)	2.9 (2.3-3.8)	2.42 (2.24-2.6)
Palau	0.7 (0.6-1)	7.9 (6.1-10.2)	2.2 (1.7-2.8)	11.4 (8.8-14.4)	1.1 (0.97-1.24)
Palestine	27.8 (17.4-40.2)	3.3 (2.1-4.8)	149.3 (124.5-177.9)	6.7 (5.6-8)	2.41 (2.15-2.67)
Panama	35.1 (32-38.4)	2.4 (2.2-2.6)	193.2 (148.5-248.9)	4.7 (3.6-6)	1.98 (1.71-2.25)
Papua New Guinea	21.7 (13.5-32.7)	1.2 (0.8-1.8)	78.9 (54.6-113.7)	1.7 (1.2-2.4)	1.15 (1.07-1.23)
Paraguay	46.6 (40.9-53.1)	2.2 (1.9-2.5)	322.7 (245-410)	6 (4.5-7.5)	3.6 (3.09-4.1)

Peru	228.3 (195.1-268.1)	2 (1.7-2.3)	1648 (1237.4-2154.9)	5.2 (3.9-6.8)	4.08 (3.34-4.82)
Philippines	946.9 (841.1-1061.5)	3.2 (2.9-3.6)	3207.5 (2576.8-3967.9)	4.2 (3.4-5.2)	0.55 (0.26-0.85)
Poland	3992.3 (3859.1-4107.3)	9.2 (8.8-9.4)	6908.3 (5856.7-8098.7)	9.9 (8.4-11.7)	0.11 (0.04-0.18)
Portugal	962.2 (912-1008.1)	7 (6.6-7.3)	1681.3 (1310.3-2137.8)	7 (5.5-9)	0.1 (0.03-0.18)
Puerto Rico	76.8 (70.7-82.6)	2.1 (1.9-2.3)	431.3 (332-551.2)	6 (4.6-7.7)	2.63 (1.82-3.45)
Qatar	4.3 (3.2-5.6)	4.6 (3.4-5.9)	49.8 (35.5-68.9)	7.6 (5.8-10)	1.93 (1.58-2.29)
Republic of Korea	2234.4 (2131.6-2357)	7.6 (7.3-8.1)	7982.2 (6671.2-9525.3)	9 (7.5-10.7)	0.03 (-0.21-0.26)
Republic of Moldova	290.8 (267.2-318.5)	6.5 (5.9-7.1)	457.6 (395.7-528.1)	7.9 (6.9-9.1)	1.21 (0.7-1.71)
Romania	1828.2 (1747.5-1912.2)	6.4 (6.2-6.7)	3567.6 (2912.2-4378)	9.9 (8.1-12.1)	1.19 (1.01-1.37)
Russian Federation	13565.5 (13166-14094.9)	7.4 (7.2-7.7)	18503.7 (16286.8-21061.3)	7.9 (6.9-9)	-0.15 (-0.42-0.12)
Rwanda	83.7 (60.9-107.9)	2.9 (2.2-3.7)	193.9 (149.9-253)	3.4 (2.7-4.3)	0.15 (-0.07-0.38)
Saint Kitts and Nevis	0.8 (0.7-0.8)	2 (1.8-2.2)	5.7 (4.7-6.8)	9.1 (7.6-10.8)	5.12 (3.9-6.36)
Saint Lucia	1.6 (1.5-1.7)	1.9 (1.7-2)	15.6 (13.1-18.5)	7.3 (6.2-8.7)	4.19 (3.28-5.11)
Saint Vincent and the Grenadines	1.2 (1.1-1.3)	1.6 (1.5-1.8)	8.2 (7.1-9.5)	6.2 (5.3-7.1)	4.05 (3.39-4.71)
Samoa	3.5 (2.9-4.2)	4 (3.4-4.9)	6 (4.8-7.8)	4.2 (3.4-5.4)	-0.35 (-0.59--0.11)
San Marino	2.9 (2.5-3.3)	8.7 (7.5-9.9)	7.2 (5.5-9.5)	10.9 (8.3-14.5)	1.09 (0.98-1.2)
Sao Tome and Principe	0.9 (0.7-1)	1.3 (1.1-1.6)	3 (2-4.2)	3 (2-4.1)	2.78 (2.73-2.84)
Saudi Arabia	86.9 (60.3-116.4)	1.6 (1.1-2.1)	889.6 (685-1142.1)	5 (4.1-6.1)	3.57 (3.07-4.08)
Senegal	73 (59-85.5)	2.3 (1.9-2.7)	325.1 (259.3-405.3)	4.5 (3.7-5.6)	2.44 (2.26-2.62)
Serbia	741.3 (652-839.1)	6.5 (5.8-7.3)	1631 (1283-2037.6)	10.2 (8.1-12.8)	1.63 (1.48-1.77)
Seychelles	2.9 (2.6-3.3)	5.2 (4.5-5.8)	7.7 (6.7-8.9)	7.2 (6.3-8.3)	1.13 (0.98-1.27)
Sierra Leone	35 (27.6-43.1)	1.9 (1.5-2.3)	119.7 (93.2-154.1)	3.5 (2.7-4.4)	2.37 (2.3-2.44)
Singapore	115.4 (107.1-124.2)	5.4 (5-5.9)	462.6 (369.7-571.8)	6.1 (4.9-7.5)	0.64 (0.49-0.78)
Slovakia	551 (523.3-581.5)	9.2 (8.7-9.7)	1061.2 (833.4-1327.8)	11.5 (9-14.4)	1 (0.84-1.17)
Slovenia	196 (151.5-250.4)	8 (6.2-10.2)	415.6 (326.3-534.4)	9.5 (7.4-12.4)	0.8 (0.6-0.99)
Solomon Islands	2.5 (1.8-3.6)	1.8 (1.3-2.4)	9.4 (6.1-12.9)	3 (2.1-3.9)	1.54 (1.28-1.79)
Somalia	44.1 (27.4-66.3)	1.7 (1.1-2.6)	104.4 (55.2-167.3)	1.6 (0.9-2.5)	-0.21 (-0.25--0.17)
South Africa	917.5 (777.9-1132.7)	4.5 (3.8-5.6)	2527.6 (2251.8-2851.3)	5.9 (5.3-6.6)	0.73 (0.43-1.03)
South Sudan	57.2 (41.1-84.9)	2.4 (1.8-3.6)	101.7 (71.9-144.9)	2.8 (2-3.9)	0.38 (0.33-0.44)
Spain	3850.8 (3649.6-4023)	7.1 (6.7-7.4)	8035 (6254-10037.8)	8.4 (6.5-10.5)	0.87 (0.73-1.02)
Sri Lanka	179.3 (161.3-198.1)	1.7 (1.5-1.9)	716.8 (535.6-953.4)	2.9 (2.1-3.8)	2.37 (2.2-2.55)
Sudan	121.8 (78.6-193.1)	1.3 (0.8-2.1)	646.6 (431.6-998.5)	3.6 (2.4-5.5)	3.64 (3.28-3.99)
Suriname	4.1 (3.7-4.5)	1.6 (1.5-1.8)	41.4 (34.1-49.8)	7 (5.7-8.3)	4.79 (3.99-5.59)
Sweden	1424.6 (1334.9-1495.7)	9.4 (8.9-9.8)	1777.6 (1485-2052.5)	8.4 (7.1-9.7)	-0.65 (-0.89--0.41)
Switzerland	682.7 (637.8-723.3)	6.5 (6.1-6.8)	1672.8 (1301.1-2107.5)	9.4 (7.3-11.9)	1.21 (0.94-1.48)
Syrian Arab Republic	90.1 (67.1-116.6)	1.7 (1.3-2.3)	430.6 (318.9-590.9)	3.6 (2.7-4.9)	2.79 (2.6-2.97)

Taiwan (Province of China)	677.3 (650.4-704.9)	4.2 (4.1-4.4)	3377.3 (2618.3-4397.1)	8.5 (6.6-11.1)	2.61 (2.41-2.8)
Tajikistan	91.1 (63.8-133.5)	3.4 (2.3-5)	217.9 (178.1-269.1)	5.2 (4.3-6.4)	1.61 (1.31-1.9)
Thailand	1119.4 (989.5-1270.6)	3.2 (2.8-3.6)	3998.9 (2986-5156.1)	4 (3-5.1)	0.04 (-0.26-0.33)
Timor-Leste	4.4 (2.9-6)	1.6 (1.1-2.1)	22.7 (16.2-29.4)	2.9 (2-3.7)	2.58 (2.22-2.94)
Togo	27.6 (22-34.1)	2.3 (1.9-2.8)	158.6 (113.8-215.5)	4.6 (3.4-6.2)	2.27 (2.14-2.41)
Tokelau	0 (0-0)	2.5 (1.9-3.2)	0.1 (0-0.1)	4.3 (3-5.5)	1.81 (1.71-1.91)
Tonga	1.5 (1.2-1.9)	2.8 (2.2-3.5)	3.3 (2.4-4.4)	4.3 (3.1-5.6)	1 (0.54-1.46)
Trinidad and Tobago	11.3 (10.4-12.4)	1.4 (1.3-1.5)	104.4 (78.5-136.5)	5.7 (4.3-7.4)	5.31 (4.21-6.42)
Tunisia	96.1 (73.6-118.7)	2 (1.5-2.4)	465.1 (343.8-619.5)	3.8 (2.8-5)	2.19 (2.15-2.22)
Turkey	1806.8 (1473.5-2229.5)	5.1 (4.1-6.2)	7064.7 (5643-8691.8)	8.1 (6.5-9.9)	1.91 (1.52-2.29)
Turkmenistan	25.1 (22.9-27.6)	1.4 (1.2-1.5)	140.7 (113-177.4)	3.7 (3-4.6)	3.73 (3.54-3.93)
Tuvalu	0.2 (0.1-0.2)	2.5 (2-3)	0.4 (0.3-0.5)	3.8 (2.8-5.1)	1.25 (1.08-1.43)
Uganda	146.5 (116.4-176.3)	2.3 (1.9-2.7)	648.1 (500.8-813.5)	4.7 (3.7-5.9)	2.52 (2.36-2.69)
Ukraine	3569.4 (2956.1-4243.9)	5 (4.1-5.9)	6067.1 (5103.3-7104.3)	8.3 (6.9-9.7)	1.38 (1.11-1.64)
United Arab Emirates	34.3 (19.2-60.8)	7.8 (4.1-13.9)	761.2 (383.1-1207.6)	17 (9.1-25.4)	2.73 (2.26-3.2)
United Kingdom	7807.9 (7493.2-7992.7)	8.5 (8.2-8.7)	11862.3 (9899.4-13881.1)	9.2 (7.7-10.8)	0.27 (0.22-0.32)
United Republic of Tanzania	275.5 (226-340.2)	2.6 (2.1-3.1)	864.4 (670.6-1122.9)	3.6 (2.9-4.6)	1.16 (1.08-1.24)
United States of America	28915.3 (27527.7-29709.6)	9 (8.6-9.2)	58421.8 (50441-67306.5)	10.4 (8.9-12)	0.52 (0.49-0.55)
United States Virgin Islands	3.1 (2.6-3.6)	3.7 (3.2-4.3)	20 (16.5-23.9)	10.9 (8.9-13)	4.23 (3.74-4.72)
Uruguay	395 (362.5-428.1)	10.1 (9.3-10.9)	764.1 (605.1-950.6)	13.9 (11-17.4)	1.16 (1.08-1.24)
Uzbekistan	166.1 (128.6-250.5)	1.5 (1.1-2.3)	849.4 (709.2-1012.4)	4.8 (4.1-5.6)	4.41 (4.19-4.63)
Vanuatu	1.1 (0.8-1.6)	1.8 (1.3-2.5)	5 (3.9-6.6)	3 (2.3-3.8)	1.61 (1.5-1.73)
Venezuela (Bolivarian Republic of)	141.3 (131-152.1)	1.5 (1.4-1.6)	1625.1 (1222.9-2115.9)	5.7 (4.3-7.3)	3.71 (2.72-4.71)
Viet Nam	672.5 (551.4-799.3)	1.7 (1.4-2)	3824.6 (2971.8-4869.6)	4.3 (3.4-5.4)	3.82 (3.59-4.04)
Yemen	57.3 (32.5-91.3)	1.2 (0.7-1.9)	273.7 (203.2-365)	2.1 (1.6-2.8)	2.45 (2.32-2.59)
Zambia	92.8 (74.7-112.3)	3.3 (2.7-4)	313.2 (220.3-438)	4.8 (3.4-6.6)	0.97 (0.85-1.08)
Zimbabwe	183.6 (159.8-210.2)	4.8 (4.2-5.4)	517.7 (395.1-660.2)	7.7 (5.9-9.7)	1.29 (0.88-1.7)

location	Cases in 1990	ASMR in 1990	Cases in 2019	ASMR in 2019	EAPC (95% CI)
Afghanistan	137.9 (82.7-235.5)	2 (1.2-3.3)	318.8 (213.2-476.5)	2.7 (1.9-3.9)	1.38 (1.1-1.66)
Albania	76 (70.1-81.8)	3.8 (3.5-4.1)	288.1 (217.1-375.7)	6.7 (5-8.7)	2.53 (2.26-2.8)
Algeria	241 (193.6-298.1)	2.2 (1.8-2.7)	1319.3 (1053.9-1615.1)	4.3 (3.5-5.2)	2.28 (2.24-2.33)
American Samoa	0.7 (0.6-0.8)	3.3 (2.7-3.8)	2.4 (2-2.8)	5.2 (4.3-6.2)	1.64 (1.23-2.06)
Andorra	4.9 (3.7-6.7)	9.4 (7.2-12.7)	14.7 (11.3-18.8)	10.3 (7.8-13.1)	0.25 (0.22-0.28)
Angola	80.3 (56.6-109.2)	2.2 (1.6-2.9)	346.1 (272.8-447.1)	3.4 (2.7-4.3)	1.37 (1.2-1.55)
Antigua and Barbuda	0.8 (0.7-0.9)	1.4 (1.3-1.6)	6.2 (5.2-7.2)	6.4 (5.4-7.5)	4.93 (4.02-5.84)

Argentina	3036 (2738.5-3349.4)	9.6 (8.6-10.5)	6513.4 (5940.3-7109.2)	11.9 (10.9-13)	0.58 (0.38-0.77)
Armenia	178.2 (151.2-208.5)	6.9 (5.8-8.1)	437.7 (363.9-521.7)	10.6 (8.8-12.6)	1.59 (1.33-1.85)
Australia	1476.3 (1404.1-1530.5)	7.5 (7.2-7.8)	3561 (3177.3-3940.6)	8.3 (7.4-9.1)	0.33 (0.26-0.4)
Austria	1271.7 (1208.9-1324.7)	10.6 (10.1-11)	1905.6 (1728.3-2075.3)	10.3 (9.5-11.2)	-0.1 (-0.18--0.02)
Azerbaijan	171.6 (149.3-202.7)	3.5 (3-4.2)	629.6 (533.4-765.7)	7.5 (6.3-9.1)	3.1 (2.81-3.38)
Bahamas	2.8 (2.5-3.1)	1.9 (1.7-2.1)	17.7 (14.4-21.8)	4.7 (3.8-5.7)	3.43 (2.71-4.15)
Bahrain	10 (8.5-11.9)	6.8 (5.8-7.9)	57.6 (44.7-72.6)	7.6 (6.1-9.2)	0.03 (-0.29-0.35)
Bangladesh	609.9 (382.7-849.4)	1.4 (0.9-2)	2734.3 (1714.4-4080.2)	2.2 (1.4-3.3)	1.61 (1.5-1.73)
Barbados	4.4 (4-4.9)	1.5 (1.4-1.6)	40.3 (32.8-47.8)	8.1 (6.6-9.7)	6.04 (4.76-7.34)
Belarus	648.6 (603.6-696.9)	5 (4.7-5.4)	1121.6 (880-1439.6)	7.1 (5.5-9.1)	1.06 (0.9-1.21)
Belgium	1446.8 (1356.4-1523.7)	9.3 (8.7-9.7)	2205 (1977.5-2424.6)	9.3 (8.4-10.1)	0.14 (-0.17-0.45)
Belize	1.5 (1.3-1.7)	1.7 (1.5-1.8)	16.9 (14.1-19.7)	6.4 (5.3-7.4)	4.45 (3.19-5.72)
Benin	44.1 (37.6-51.7)	2.3 (2-2.7)	220.7 (171.6-282.6)	4.9 (3.9-6.2)	2.76 (2.52-3.01)
Bermuda	1.8 (1.6-2)	3 (2.7-3.3)	12.9 (10.5-15.7)	9.6 (7.9-11.7)	3.72 (2.55-4.91)
Bhutan	3.2 (1.6-5)	1.5 (0.7-2.3)	17.2 (9.8-26.2)	3.3 (1.9-5)	3.04 (2.95-3.12)
Bolivia (Plurinational State of)	77 (56.2-97.7)	2.5 (1.9-3.2)	445.3 (304.7-599.2)	5.3 (3.7-7.2)	2.43 (2.24-2.62)
Bosnia and Herzegovina	301.1 (282.8-320.3)	7.8 (7.3-8.3)	603 (482.1-747.2)	10.1 (8.1-12.5)	0.8 (0.71-0.9)
Botswana	20 (15.7-25.4)	3.8 (3.1-4.7)	97.2 (69.8-133.6)	8 (5.9-10.6)	2.2 (1.91-2.5)
Brazil	4552 (4350.9-4703.3)	5.5 (5.2-5.7)	14977.3 (13838.1-15869.4)	6.4 (5.9-6.8)	0.63 (0.57-0.7)
Brunei Darussalam	5.6 (4.7-6.6)	6.6 (5.6-7.7)	23 (19.8-26.3)	9.3 (8.1-10.6)	1.55 (1.38-1.73)
Bulgaria	854.9 (804.3-906.9)	6.9 (6.5-7.3)	1574 (1248.5-1961.6)	11.1 (8.8-13.9)	3 (2.51-3.49)
Burkina Faso	75.5 (58.3-96.7)	1.8 (1.4-2.3)	304 (230.3-386)	3.6 (2.8-4.6)	2.4 (2.34-2.46)
Burundi	54.2 (41.3-68.2)	2.4 (1.9-2.9)	98.6 (71.5-137.8)	2.3 (1.7-3.2)	-0.32 (-0.41--0.22)
Cabo Verde	2.9 (2.6-3.3)	1.2 (1.1-1.4)	44.2 (34.6-53.7)	10.8 (8.4-13.2)	6.64 (5.39-7.9)
Cambodia	95.6 (71-124.4)	2.2 (1.7-2.9)	418 (333.8-502.6)	3.7 (3-4.4)	1.92 (1.81-2.04)
Cameroon	161 (125.5-198.2)	3.9 (3-4.8)	866.4 (605.2-1190.7)	8 (5.7-10.8)	2.43 (2.32-2.54)
Canada	2775 (2633.4-2878.1)	8.6 (8.1-8.9)	6054.8 (5474.4-6647.1)	8.6 (7.8-9.4)	-0.01 (-0.08-0.06)
Central African Republic	24.5 (16.6-33.6)	2.2 (1.6-2.9)	45.6 (29.9-63.3)	2.2 (1.6-2.9)	0.03 (-0.02-0.08)
Chad	38.4 (29.9-48)	1.4 (1.1-1.8)	147.4 (117.1-184.9)	2.9 (2.3-3.6)	2.54 (2.38-2.7)
Chile	666 (623.2-710.7)	7 (6.5-7.5)	1873.6 (1689.1-2056.7)	7.8 (7-8.5)	0.42 (0.36-0.47)
China	27104.2 (23604.4-30844.3)	3.3 (2.9-3.8)	117374 (99862.7-136452.7)	6 (5.1-6.9)	2.25 (2.06-2.44)
Colombia	911.5 (864.4-957.2)	5.5 (5.2-5.8)	2495.5 (1919.6-3167.4)	4.7 (3.6-6)	-0.75 (-0.98--0.52)
Comoros	6.2 (3.7-7.9)	2.9 (1.8-3.7)	18.1 (14-23)	3.9 (3-4.9)	0.79 (0.65-0.92)
Congo	39.9 (26.9-54.2)	3.9 (2.8-5.1)	131.8 (84.4-191)	5.5 (3.6-7.7)	0.96 (0.72-1.2)
Cook Islands	0.5 (0.4-0.6)	3.8 (3.1-4.6)	1.2 (1-1.5)	4.9 (4.2-5.9)	0.62 (0.46-0.78)
Costa Rica	54.2 (49.4-59.1)	3.2 (2.9-3.5)	367.2 (284.6-461.4)	7.2 (5.6-9.1)	2.45 (1.9-3.01)

Croatia	530.6 (495.3-565.9)	8.4 (7.9-9)	813.2 (650.5-1019.2)	9.1 (7.2-11.4)	0.63 (0.46-0.8)
Cuba	178.6 (165.9-191)	1.7 (1.6-1.9)	1186.8 (967.4-1449.8)	6.2 (5-7.5)	4.29 (3.24-5.35)
Cyprus	33.2 (29.5-37.1)	4.3 (3.9-4.8)	156.7 (135.5-179)	8.2 (7.1-9.4)	2.8 (2.45-3.15)
Czechia	1644.4 (1574.7-1710.1)	11.9 (11.4-12.4)	2529.6 (2044.4-3053.5)	11.8 (9.6-14.3)	0.19 (0.02-0.36)
C么te d'Ivoire	103.2 (82.1-128.1)	2.8 (2.3-3.4)	518.2 (396.6-655.9)	5.5 (4.3-6.8)	1.97 (1.74-2.21)
Democratic People's Republic of Korea	473.2 (361.5-618.2)	3 (2.3-3.8)	1241.3 (941-1570.6)	3.9 (3-4.8)	1.03 (0.97-1.08)
Democratic Republic of the Congo	316.1 (247.9-402.9)	2.2 (1.7-2.8)	793.6 (600-1029.7)	2.4 (1.8-3.1)	-0.04 (-0.4-0.32)
Denmark	617.9 (582.3-648.3)	7.5 (7.1-7.8)	1193.6 (1061.2-1319.4)	10.1 (9-11.1)	0.91 (0.53-1.29)
Djibouti	3.3 (2.4-4.5)	2.6 (1.9-3.4)	23.5 (16.1-33.3)	4.3 (3.1-5.8)	1.8 (1.71-1.89)
Dominica	1.4 (1.2-1.5)	1.9 (1.7-2.1)	7.7 (6.2-9.4)	8.5 (6.8-10.4)	5.47 (4.52-6.42)
Dominican Republic	52.6 (46.1-60)	1.5 (1.3-1.7)	338.5 (231.6-458.9)	3.7 (2.6-5)	4.09 (3.6-4.59)
Ecuador	97.4 (91.1-104)	1.9 (1.8-2.1)	839.9 (669.5-1058.8)	5.8 (4.6-7.3)	3.55 (2.8-4.29)
Egypt	592.2 (534.5-642.6)	2.1 (1.9-2.3)	2768 (1899.5-3877.1)	4.4 (3.1-6.1)	2.43 (2.24-2.61)
El Salvador	50.9 (46.9-55.1)	1.8 (1.6-1.9)	314.7 (236.8-404.2)	5.3 (3.9-6.8)	3.89 (3.28-4.51)
Equatorial Guinea	3.7 (2.4-5.5)	1.9 (1.3-2.8)	27.4 (16.3-42)	6.4 (4-9.4)	4.94 (4.69-5.19)
Eritrea	17.4 (12.6-23.2)	1.8 (1.3-2.4)	77.3 (51.7-108.2)	3.1 (2.2-4.3)	1.59 (1.31-1.87)
Estonia	177.3 (165.4-189)	8.7 (8.1-9.2)	278 (218.4-346.9)	10.2 (8-12.9)	0.66 (0.42-0.91)
Eswatini	13 (9.7-17.1)	4.8 (3.7-6.3)	43.3 (29.3-60.3)	8.2 (5.7-11.2)	1.38 (0.71-2.06)
Ethiopia	233.8 (134.8-369)	1.3 (0.7-2)	602.5 (405.1-872.9)	1.6 (1.1-2.3)	0.81 (0.6-1.03)
Fiji	9 (7.3-10.9)	2.7 (2.2-3.3)	29.5 (23-37.1)	4.3 (3.4-5.4)	1.51 (1.44-1.58)
Finland	763.7 (724.4-796.7)	10.6 (10-11)	1377.7 (1219-1527.6)	10.7 (9.6-11.8)	0.26 (0.15-0.37)
France	6625.7 (6252.3-6907.9)	7.9 (7.5-8.2)	14045.5 (12223.6-15608.8)	9.8 (8.7-10.7)	0.91 (0.81-1.01)
Gabon	22 (16.9-34.4)	4.1 (3.2-6.2)	79.7 (54.1-111)	8.2 (5.7-11.2)	2.17 (2.02-2.33)
Gambia	5.2 (4-6.7)	1.6 (1.2-2)	31.5 (21.6-43.4)	3.5 (2.4-4.8)	2.59 (2.4-2.77)
Georgia	237.2 (193.5-306.6)	3.9 (3.2-5)	370.7 (306.1-437.9)	6.3 (5.2-7.5)	2.63 (2.05-3.22)
Germany	10605.7 (10037.3-11071.2)	8.2 (7.8-8.6)	21507.8 (19347.1-23692.6)	10.8 (9.9-11.8)	1.02 (0.81-1.23)
Ghana	270.9 (222.1-328.7)	4.6 (3.9-5.5)	1439.1 (1093.4-1884.2)	9.6 (7.3-12.6)	2.31 (2.18-2.44)
Greece	1432.5 (1358-1501.2)	9.4 (8.9-9.8)	2463.5 (2196.6-2699.3)	10 (9-10.8)	-0.03 (-0.13-0.06)
Greenland	5.1 (4.5-5.8)	16 (14.1-17.9)	12.8 (10.3-15.2)	19.3 (15.7-22.8)	0.31 (0.02-0.61)
Grenada	1.3 (1.2-1.5)	1.8 (1.6-2)	10.5 (9.2-11.9)	9.8 (8.6-11)	6.26 (5.16-7.37)
Guam	2.9 (2.5-3.3)	4.3 (3.6-5)	9.8 (8.1-11.7)	5.3 (4.4-6.3)	0.98 (0.75-1.2)
Guatemala	54.1 (47.1-62)	1.6 (1.4-1.8)	502.3 (391.3-630.9)	4.7 (3.7-5.9)	3.91 (3.08-4.75)
Guinea	38.1 (30.8-46.3)	1.2 (1-1.4)	99.4 (75.3-124.9)	1.9 (1.5-2.4)	1.61 (1.51-1.71)
Guinea-Bissau	10.5 (7.1-14.3)	2.7 (1.9-3.6)	31.5 (21-42.9)	4.7 (3.3-6.3)	2 (1.86-2.13)

Guyana	6.5 (5.6-7.5)	1.8 (1.6-2.1)	36.8 (28.1-46.8)	6.1 (4.7-7.7)	4.08 (3.24-4.92)
Haiti	38.7 (29.5-48.2)	1.3 (1-1.5)	165.5 (112.3-231.4)	2.5 (1.8-3.5)	2.71 (2.52-2.9)
Honduras	40.5 (32.4-47.5)	2.1 (1.7-2.5)	256.9 (155.5-369.9)	4.6 (2.8-6.5)	2.89 (2.74-3.04)
Hungary	1477.6 (1413.2-1547)	10.1 (9.7-10.6)	2331.2 (1922.7-2789.4)	12 (9.9-14.4)	0.61 (0.47-0.75)
Iceland	25.4 (23.2-27.7)	8.8 (8.1-9.6)	46.3 (39.9-52.8)	8.1 (7.1-9.2)	-0.27 (-0.39--0.15)
India	6168.7 (5023-7322.1)	1.5 (1.2-1.8)	33546.5 (29137.6-38690.4)	3.1 (2.7-3.6)	2.47 (2.33-2.6)
Indonesia	2180.7 (1803.9-2549.1)	2.4 (2-2.7)	10588.6 (6930.8-14830.9)	5.4 (3.5-7.5)	2.93 (2.88-2.98)
Iran (Islamic Republic of)	534.3 (433.6-645.5)	2.3 (1.8-2.7)	3360.7 (3124-3643.5)	4.9 (4.5-5.3)	2.81 (2.64-2.98)
Iraq	235.7 (163-318)	3.2 (2.2-4.3)	1329 (1007.4-1654.7)	6.1 (4.7-7.5)	2.56 (2.21-2.91)
Ireland	404.3 (382.9-426.3)	9.8 (9.3-10.4)	699.4 (617.6-778.5)	9.2 (8.1-10.2)	-0.21 (-0.27--0.15)
Israel	470 (441.3-497.8)	9.7 (9.1-10.3)	1273.1 (1135.3-1387.4)	10.7 (9.6-11.7)	0.2 (0.12-0.28)
Italy	8000.5 (7726.8-8186.2)	8.9 (8.6-9.1)	14418.8 (12787.7-15411.4)	9.6 (8.7-10.2)	0.24 (0.14-0.33)
Jamaica	25.1 (22.8-27.5)	1.4 (1.3-1.5)	154.5 (121.6-191.9)	5.1 (4-6.4)	4.3 (3.39-5.22)
Japan	15361.1 (14605.9-15775.1)	9.2 (8.7-9.4)	37462.4 (31495.5-40785.5)	9.6 (8.4-10.3)	0.34 (0.24-0.44)
Jordan	32.5 (27.3-38.9)	2.7 (2.3-3.2)	315.3 (262.1-374.2)	5.4 (4.5-6.4)	2.79 (2.62-2.96)
Kazakhstan	151.1 (135.5-172.8)	1.3 (1.1-1.4)	1129 (962.2-1311.6)	6.7 (5.7-7.7)	7.52 (6.41-8.65)
Kenya	114.8 (75.4-167.1)	1.5 (1-2.2)	695.7 (536.7-907.1)	3.4 (2.7-4.4)	3.3 (3.1-3.49)
Kiribati	0.8 (0.7-1)	2.4 (1.9-2.8)	1.8 (1.3-2.3)	3 (2.3-3.9)	0.33 (0.01-0.64)
Kuwait	21.3 (18.7-24.1)	4.1 (3.5-4.6)	121.1 (98.1-147.7)	5.6 (4.5-6.8)	1.68 (1.3-2.07)
Kyrgyzstan	113.3 (99.3-129.3)	3.8 (3.3-4.3)	232.2 (199.3-266.5)	5.3 (4.6-6.1)	1.27 (1.08-1.45)
Lao People's Democratic Republic	48.6 (34.3-68.2)	2.4 (1.7-3.3)	143.2 (111-182.3)	3.5 (2.8-4.4)	1.29 (1.2-1.38)
Latvia	292.4 (272.2-314.2)	8.2 (7.6-8.8)	411.1 (340.1-501.1)	10.2 (8.4-12.4)	0.51 (0.22-0.79)
Lebanon	71.5 (58.6-85.9)	3.3 (2.7-4)	342.3 (265.3-421.5)	6.6 (5.1-8.1)	2.79 (2.6-2.99)
Lesotho	25.4 (18.8-33.8)	2.7 (2.1-3.6)	70.9 (49.2-96.4)	6.1 (4.3-8.1)	2.96 (2.78-3.13)
Liberia	28.6 (22.9-35.6)	2.7 (2.1-3.3)	93.3 (66.2-124.3)	5.1 (3.7-6.7)	2.93 (2.53-3.33)
Libya	70.7 (44.7-98.1)	4 (2.5-5.6)	351.4 (268.9-453)	7.3 (5.6-9.3)	2.23 (2.08-2.38)
Lithuania	342.2 (322.3-362.2)	7.6 (7.1-8)	532.6 (434.8-650.8)	9.2 (7.4-11.3)	0.55 (0.32-0.78)
Luxembourg	54.4 (50.8-58.3)	9.9 (9.3-10.6)	93.4 (80.7-106.4)	9.1 (7.9-10.3)	-0.21 (-0.39--0.02)
Madagascar	98.5 (77.7-116.6)	2 (1.6-2.3)	245.4 (173.4-331.4)	2.4 (1.7-3.2)	0.55 (0.45-0.65)
Malawi	72.9 (60.6-86.2)	2.1 (1.7-2.4)	206.9 (159.2-261.6)	3.1 (2.4-3.9)	1.44 (1.34-1.55)
Malaysia	167.1 (142.4-195.1)	1.9 (1.6-2.3)	949.2 (736-1194.3)	3.8 (3-4.8)	2.44 (2.05-2.83)
Maldives	2.4 (1.6-3.4)	3 (2.2-4.1)	12.2 (10.1-14.6)	4.5 (3.7-5.4)	1.06 (0.89-1.24)
Mali	87.9 (74.8-103.3)	2.2 (1.9-2.6)	278.8 (208.3-364.5)	3.5 (2.6-4.4)	1.55 (1.5-1.59)
Malta	35.9 (32.5-39.2)	8.5 (7.7-9.2)	82.4 (69.4-95.6)	8.7 (7.3-10)	0.32 (0.18-0.46)
Marshall Islands	0.4 (0.3-0.4)	2.4 (2-2.9)	1.3 (0.9-1.7)	4 (3-5.2)	1.72 (1.64-1.81)
Mauritania	34.7 (25.6-45)	3.6 (2.7-4.6)	125.1 (90.4-164)	6.5 (4.8-8.3)	2.07 (2.01-2.13)

Mauritius	30.4 (28.2-32.8)	4.3 (4-4.6)	91.8 (73.5-112.4)	5.3 (4.3-6.5)	0.73 (0.55-0.92)
Mexico	2313.2 (2221.8-2381.5)	5.7 (5.5-5.9)	6853.3 (5866.5-7852.9)	6 (5.2-6.9)	-0.19 (-0.31--0.07)
Micronesia (Federated States of)	1.4 (1.1-1.8)	3.2 (2.6-4)	3.4 (2.4-4.8)	5.3 (3.7-7.1)	1.53 (1.29-1.77)
Monaco	9.2 (7.2-11)	12.5 (9.8-15.2)	17.9 (14.3-21.4)	17.8 (14.2-21.2)	1.44 (1.19-1.7)
Mongolia	32.2 (26-38.5)	3.2 (2.6-3.8)	137.5 (106-176.8)	6.3 (5-7.9)	2.28 (2.11-2.45)
Montenegro	44.7 (38.8-51.9)	7.3 (6.3-8.4)	107.7 (90.1-127.7)	10.9 (9.1-12.9)	1.71 (1.63-1.8)
Morocco	254.6 (205.1-309.3)	2 (1.6-2.4)	1167.8 (840.9-1516.9)	4 (2.8-5.2)	2.38 (2.17-2.6)
Mozambique	103.2 (85.5-121.8)	1.9 (1.6-2.3)	381.7 (265.6-545.6)	3.9 (2.7-5.5)	2.83 (2.69-2.98)
Myanmar	544.4 (393.9-717.6)	2.4 (1.8-3.2)	1721.7 (1348.7-2222.9)	4 (3.1-5.1)	1.85 (1.75-1.95)
Namibia	12.9 (10.4-15.6)	1.9 (1.5-2.3)	53.6 (41.6-69.3)	4.1 (3.2-5.2)	2.81 (2.68-2.94)
Nauru	0.1 (0.1-0.2)	4.4 (3.3-5.7)	0.2 (0.1-0.3)	6.1 (4.3-8.2)	0.73 (0.53-0.92)
Nepal	95.4 (53.1-142.8)	1.1 (0.6-1.7)	613.1 (367.6-897.9)	3 (1.8-4.4)	3.71 (3.43-4)
Netherlands	1773.6 (1677-1846.3)	8.8 (8.3-9.2)	3913 (3517.3-4307.1)	11.1 (10-12.2)	1.11 (0.94-1.29)
New Zealand	283.2 (265.5-301.6)	7.2 (6.7-7.6)	597.2 (539.9-653.5)	7.5 (6.8-8.1)	0.22 (0.08-0.37)
Nicaragua	35.4 (29.1-42.9)	2.5 (2-3.1)	238.5 (197.2-281.3)	6 (5-7)	2.99 (2.55-3.42)
Niger	38.1 (27.1-49.3)	1.5 (1-1.9)	164.6 (109.4-234.8)	2.4 (1.6-3.3)	1.67 (1.52-1.81)
Nigeria	880.2 (631-1143.2)	2.2 (1.6-2.8)	3217.3 (2468.3-4013.9)	4.3 (3.3-5.2)	2.4 (2.3-2.49)
Niue	0.1 (0.1-0.1)	3.9 (3-4.7)	0.1 (0.1-0.2)	6.4 (4.9-8.1)	1.66 (1.47-1.85)
North Macedonia	123.3 (113.4-134.3)	6.7 (6.2-7.3)	342.4 (272.2-429)	10.8 (8.6-13.4)	1.73 (1.59-1.86)
Northern Mariana Islands	0.5 (0.4-0.7)	3.6 (3-4.3)	3.7 (3.1-4.3)	7.8 (6.6-9)	3.39 (2.74-4.04)
Norway	605.3 (572.9-629)	8.6 (8.2-8.9)	896.8 (816.9-959.6)	9 (8.3-9.6)	0.34 (0.23-0.46)
Oman	12.8 (8.3-18.5)	2.2 (1.4-3.1)	85.9 (72.4-104.2)	6.2 (5.4-7.2)	4 (3.68-4.31)
Pakistan	858.9 (716.2-1005.8)	1.6 (1.3-1.9)	3100.9 (2403.8-3981.8)	3.1 (2.4-4)	2.39 (2.21-2.57)
Palau	0.8 (0.6-1)	8.4 (6.4-10.8)	2.2 (1.7-2.8)	12 (9.2-15)	1.06 (0.93-1.19)
Palestine	28.7 (18-41.4)	3.5 (2.2-5)	149.9 (125.5-178.5)	7 (5.8-8.3)	2.37 (2.12-2.62)
Panama	36.4 (33.1-40)	2.5 (2.3-2.8)	202.1 (154.5-259.8)	4.9 (3.7-6.3)	1.97 (1.7-2.25)
Papua New Guinea	21.7 (13.6-32.8)	1.3 (0.8-1.9)	78.8 (54.7-112.9)	1.8 (1.3-2.6)	1.15 (1.07-1.23)
Paraguay	48.8 (42.8-55.7)	2.3 (2-2.6)	335.4 (255.5-423.1)	6.3 (4.8-7.9)	3.56 (3.04-4.08)
Peru	237.8 (203.1-278.5)	2.1 (1.8-2.4)	1722.2 (1303.5-2249.9)	5.4 (4.1-7.1)	4.02 (3.28-4.77)
Philippines	958.7 (848.8-1078.2)	3.4 (3-3.8)	3249.6 (2654.3-3957.6)	4.4 (3.6-5.3)	0.53 (0.23-0.82)
Poland	4220.2 (4066-4343.7)	9.8 (9.4-10.1)	7446.9 (6268.6-8799.5)	10.6 (8.9-12.5)	0.1 (0.03-0.16)
Portugal	1027.3 (973.1-1080.9)	7.5 (7.1-7.9)	1842.5 (1627.2-2031.2)	7.4 (6.6-8.1)	0.05 (-0.02-0.12)
Puerto Rico	79.9 (73.4-86.3)	2.2 (2-2.4)	450.4 (346.3-574.2)	6.1 (4.7-7.8)	2.51 (1.7-3.33)
Qatar	4.2 (3.1-5.5)	4.9 (3.6-6.4)	46.7 (33.5-64.4)	8.1 (6.1-10.5)	1.9 (1.56-2.24)
Republic of Korea	2197.5 (2097.8-2321.3)	7.7 (7.3-8.2)	7303.1 (6519.3-8149.8)	8.2 (7.3-9.2)	-0.33 (-0.57--0.09)
Republic of Moldova	290.8 (267.4-318.5)	6.6 (6-7.2)	459.6 (397.4-529.8)	8 (6.9-9.1)	1.16 (0.65-1.66)

Romania	1832.8 (1749.2-1916.6)	6.5 (6.2-6.8)	3644.1 (2969-4465.4)	9.9 (8.1-12.2)	1.17 (0.99-1.36)
Russian Federation	13920.3 (13489.7-14482.3)	7.7 (7.4-8)	19377.9 (16968.1-21965.2)	8.2 (7.2-9.3)	-0.11 (-0.37-0.15)
Rwanda	85.6 (63-110.3)	3.1 (2.3-3.9)	198.9 (154.3-258.5)	3.6 (2.9-4.6)	0.18 (-0.05-0.41)
Saint Kitts and Nevis	0.8 (0.7-0.9)	2.1 (1.9-2.3)	5.8 (4.8-6.8)	9.5 (8-11.2)	5.16 (3.94-6.39)
Saint Lucia	1.7 (1.5-1.8)	2 (1.8-2.2)	16.3 (13.7-19.2)	7.7 (6.5-9.1)	4.11 (3.19-5.04)
Saint Vincent and the Grenadines	1.2 (1.1-1.3)	1.7 (1.6-1.9)	8.6 (7.4-9.8)	6.5 (5.6-7.4)	4.05 (3.4-4.7)
Samoa	3.6 (3-4.3)	4.3 (3.5-5.1)	6.2 (5-7.9)	4.4 (3.6-5.6)	-0.38 (-0.61--0.15)
San Marino	2.9 (2.5-3.4)	8.7 (7.4-10.1)	7.5 (5.1-10.6)	11.1 (7.4-16)	1.19 (1.07-1.32)
Sao Tome and Principe	0.9 (0.7-1)	1.4 (1.2-1.7)	3.1 (2.1-4.3)	3.1 (2.1-4.3)	2.78 (2.72-2.83)
Saudi Arabia	100.1 (67.2-139)	1.9 (1.2-2.6)	846.6 (655.8-1081)	5.1 (4.2-6.2)	2.84 (2.33-3.35)
Senegal	76 (61.7-88.8)	2.5 (2-2.9)	338.9 (273.4-417.8)	4.9 (4-6)	2.45 (2.27-2.62)
Serbia	764 (673.6-867.5)	6.9 (6-7.8)	1734.3 (1366.6-2156)	10.8 (8.5-13.4)	1.66 (1.52-1.81)
Seychelles	3 (2.7-3.4)	5.4 (4.8-6.1)	7.9 (6.9-9.1)	7.5 (6.5-8.6)	1.12 (0.97-1.26)
Sierra Leone	36.9 (29.3-45.3)	2 (1.6-2.4)	124.2 (97.6-159.3)	3.7 (3-4.7)	2.36 (2.3-2.43)
Singapore	113.3 (105.2-121.6)	5.4 (5-5.8)	425.3 (379.7-466.4)	5.6 (5-6.1)	0.33 (0.2-0.46)
Slovakia	507.3 (482.2-535.3)	8.5 (8.1-8.9)	964.5 (758.5-1207)	10.4 (8.2-13)	0.93 (0.77-1.09)
Slovenia	200.7 (156.6-255.7)	8.2 (6.4-10.5)	421.5 (329.8-541.4)	9.5 (7.4-12.2)	0.67 (0.47-0.86)
Solomon Islands	2.5 (1.8-3.5)	1.9 (1.4-2.5)	9.2 (6.1-12.6)	3.1 (2.2-4)	1.53 (1.27-1.78)
Somalia	44.5 (27.5-66.7)	1.8 (1.2-2.7)	107 (57.7-173)	1.7 (0.9-2.7)	-0.17 (-0.21--0.12)
South Africa	947.4 (794.4-1172)	4.8 (4-5.9)	2628.4 (2347.6-2955.5)	6.3 (5.6-7)	0.76 (0.46-1.06)
South Sudan	59.8 (43.2-88.6)	2.6 (1.9-3.9)	105.3 (73.3-151.8)	3 (2.1-4.2)	0.4 (0.35-0.44)
Spain	3827.7 (3617.5-4002.2)	7 (6.6-7.3)	7900.5 (6997.8-8687.9)	8 (7.2-8.8)	0.78 (0.62-0.94)
Sri Lanka	183.9 (165.7-202.9)	1.8 (1.6-2)	734.2 (548.5-969.9)	3 (2.2-3.9)	2.32 (2.14-2.51)
Sudan	125.4 (80.5-197.8)	1.4 (0.9-2.2)	654 (446.7-1014)	3.8 (2.6-5.8)	3.6 (3.25-3.96)
Suriname	4.3 (3.9-4.7)	1.7 (1.6-1.9)	42.7 (35.2-51.3)	7.3 (6-8.7)	4.74 (3.95-5.55)
Sweden	1559.5 (1449.6-1637.8)	10 (9.4-10.5)	1976.2 (1792.8-2128.6)	9 (8.3-9.7)	-0.65 (-0.91--0.4)
Switzerland	661.5 (616.2-699.5)	6.2 (5.8-6.6)	1581.1 (1405.9-1741.7)	8.7 (7.8-9.6)	1.11 (0.85-1.36)
Syrian Arab Republic	91.4 (67.6-118.6)	1.8 (1.3-2.4)	430.8 (319.3-587.7)	3.8 (2.8-5)	2.74 (2.55-2.92)
Taiwan (Province of China)	674.4 (647.7-702)	4.3 (4.1-4.5)	3386.9 (2637.7-4409.1)	8.5 (6.6-11.1)	2.51 (2.33-2.7)
Tajikistan	95.5 (65.6-142.2)	3.6 (2.4-5.4)	222.4 (182.1-274)	5.6 (4.7-6.9)	1.7 (1.42-1.98)
Thailand	1133.5 (1002.2-1286.4)	3.3 (3-3.8)	4140.5 (3096.4-5331.4)	4.1 (3.1-5.3)	0.01 (-0.27-0.3)
Timor-Leste	4.4 (2.9-6)	1.7 (1.1-2.3)	23.6 (16.9-30.7)	3.1 (2.2-4)	2.61 (2.26-2.96)
Togo	28.2 (22.5-34.7)	2.4 (2-3)	161.6 (116.9-217.3)	4.9 (3.6-6.5)	2.29 (2.16-2.42)
Tokelau	0 (0-0)	2.6 (2-3.4)	0.1 (0-0.1)	4.5 (3.1-5.8)	1.79 (1.69-1.89)
Tonga	1.6 (1.2-2)	3 (2.3-3.7)	3.5 (2.6-4.6)	4.5 (3.3-5.9)	0.97 (0.51-1.43)
Trinidad and Tobago	11.8 (10.8-12.9)	1.4 (1.3-1.6)	109.5 (82.8-142.4)	6 (4.6-7.8)	5.32 (4.22-6.43)

Tunisia	98.5 (75.4-121.8)	2.1 (1.6-2.6)	470.8 (350.2-623)	3.9 (2.9-5.1)	2.09 (2.06-2.13)
Turkey	1837.1 (1501.3-2261.5)	5.3 (4.3-6.5)	7132.4 (5728.7-8753.6)	8.2 (6.6-10.1)	1.83 (1.46-2.21)
Turkmenistan	25.7 (23.5-28.3)	1.4 (1.3-1.6)	141.4 (113.5-177.7)	3.9 (3.1-4.8)	3.66 (3.46-3.85)
Tuvalu	0.2 (0.1-0.2)	2.6 (2.1-3.2)	0.4 (0.3-0.5)	4 (3-5.4)	1.24 (1.06-1.41)
Uganda	152.2 (121.4-182.6)	2.5 (2-2.9)	660.9 (511.1-826.6)	5 (3.9-6.2)	2.53 (2.38-2.68)
Ukraine	3565.2 (2952.1-4230.3)	5 (4.1-5.9)	5948.6 (5022.4-7009)	8 (6.8-9.4)	1.26 (1.01-1.51)
United Arab Emirates	33 (18.3-58.5)	8.2 (4.3-14.6)	708.7 (358.8-1120)	17.6 (9.4-26.2)	2.67 (2.18-3.17)
United Kingdom	7966.6 (7631.1-8162)	8.6 (8.3-8.9)	12071.4 (11109.6-12692.1)	9.2 (8.6-9.7)	0.21 (0.16-0.26)
United Republic of Tanzania	284.7 (233.9-350)	2.8 (2.3-3.3)	892 (698.1-1151.8)	3.9 (3.1-4.9)	1.15 (1.07-1.24)
United States of America	28596 (27103.3-29427.7)	8.8 (8.4-9)	57487.9 (53665.2-60245.1)	10.1 (9.4-10.5)	0.49 (0.46-0.52)
United States Virgin Islands	3.1 (2.7-3.7)	3.9 (3.3-4.5)	20.7 (17.1-24.6)	11.3 (9.3-13.4)	4.18 (3.7-4.66)
Uruguay	412 (377.7-446.1)	10.5 (9.7-11.4)	811.5 (739.4-886)	14.5 (13.2-15.7)	1.11 (1.04-1.19)
Uzbekistan	169.5 (130.4-259.8)	1.5 (1.2-2.4)	843 (706-1004.8)	5.2 (4.4-6)	4.55 (4.31-4.78)
Vanuatu	1.2 (0.8-1.6)	1.9 (1.4-2.6)	5.1 (4-6.7)	3.1 (2.4-4)	1.59 (1.49-1.7)
Venezuela (Bolivarian Republic of)	145.1 (134.4-156.4)	1.6 (1.4-1.7)	1667.1 (1261-2156.9)	5.9 (4.4-7.5)	3.65 (2.66-4.64)
Viet Nam	705.8 (583.2-834.8)	1.8 (1.5-2.1)	3923.9 (3070-4959.7)	4.5 (3.6-5.7)	3.76 (3.54-3.98)
Yemen	58 (33.1-92.3)	1.2 (0.7-2)	277.5 (206.9-370.9)	2.2 (1.7-2.9)	2.46 (2.32-2.59)
Zambia	95.5 (77.1-116.4)	3.6 (2.9-4.3)	318.3 (225-443.9)	5.1 (3.7-6.9)	0.95 (0.84-1.06)
Zimbabwe	186 (162.8-213.3)	5.1 (4.4-5.7)	517.4 (397-658.6)	8.1 (6.2-10.2)	1.37 (1.08-1.66)

location	Cases in 1990	ASDR in 1990	Cases in 2019	ASDR in 2019	EAPC (95% CI)
Afghanistan	137.9 (82.7-235.5)	2 (1.2-3.3)	318.8 (213.2-476.5)	2.7 (1.9-3.9)	1.38 (1.1-1.66)
Albania	76 (70.1-81.8)	3.8 (3.5-4.1)	288.1 (217.1-375.7)	6.7 (5-8.7)	2.53 (2.26-2.8)
Algeria	241 (193.6-298.1)	2.2 (1.8-2.7)	1319.3 (1053.9-1615.1)	4.3 (3.5-5.2)	2.28 (2.24-2.33)
American Samoa	0.7 (0.6-0.8)	3.3 (2.7-3.8)	2.4 (2-2.8)	5.2 (4.3-6.2)	1.64 (1.23-2.06)
Andorra	4.9 (3.7-6.7)	9.4 (7.2-12.7)	14.7 (11.3-18.8)	10.3 (7.8-13.1)	0.25 (0.22-0.28)
Angola	80.3 (56.6-109.2)	2.2 (1.6-2.9)	346.1 (272.8-447.1)	3.4 (2.7-4.3)	1.37 (1.2-1.55)
Antigua and Barbuda	0.8 (0.7-0.9)	1.4 (1.3-1.6)	6.2 (5.2-7.2)	6.4 (5.4-7.5)	4.93 (4.02-5.84)
Argentina	3036 (2738.5-3349.4)	9.6 (8.6-10.5)	6513.4 (5940.3-7109.2)	11.9 (10.9-13)	0.58 (0.38-0.77)
Armenia	178.2 (151.2-208.5)	6.9 (5.8-8.1)	437.7 (363.9-521.7)	10.6 (8.8-12.6)	1.59 (1.33-1.85)
Australia	1476.3 (1404.1-1530.5)	7.5 (7.2-7.8)	3561 (3177.3-3940.6)	8.3 (7.4-9.1)	0.33 (0.26-0.4)
Austria	1271.7 (1208.9-1324.7)	10.6 (10.1-11)	1905.6 (1728.3-2075.3)	10.3 (9.5-11.2)	-0.1 (-0.18--0.02)
Azerbaijan	171.6 (149.3-202.7)	3.5 (3-4.2)	629.6 (533.4-765.7)	7.5 (6.3-9.1)	3.1 (2.81-3.38)
Bahamas	2.8 (2.5-3.1)	1.9 (1.7-2.1)	17.7 (14.4-21.8)	4.7 (3.8-5.7)	3.43 (2.71-4.15)
Bahrain	10 (8.5-11.9)	6.8 (5.8-7.9)	57.6 (44.7-72.6)	7.6 (6.1-9.2)	0.03 (-0.29-0.35)
Bangladesh	609.9 (382.7-849.4)	1.4 (0.9-2)	2734.3 (1714.4-4080.2)	2.2 (1.4-3.3)	1.61 (1.5-1.73)

Barbados	4.4 (4-4.9)	1.5 (1.4-1.6)	40.3 (32.8-47.8)	8.1 (6.6-9.7)	6.04 (4.76-7.34)
Belarus	648.6 (603.6-696.9)	5 (4.7-5.4)	1121.6 (880-1439.6)	7.1 (5.5-9.1)	1.06 (0.9-1.21)
Belgium	1446.8 (1356.4-1523.7)	9.3 (8.7-9.7)	2205 (1977.5-2424.6)	9.3 (8.4-10.1)	0.14 (-0.17-0.45)
Belize	1.5 (1.3-1.7)	1.7 (1.5-1.8)	16.9 (14.1-19.7)	6.4 (5.3-7.4)	4.45 (3.19-5.72)
Benin	44.1 (37.6-51.7)	2.3 (2-2.7)	220.7 (171.6-282.6)	4.9 (3.9-6.2)	2.76 (2.52-3.01)
Bermuda	1.8 (1.6-2)	3 (2.7-3.3)	12.9 (10.5-15.7)	9.6 (7.9-11.7)	3.72 (2.55-4.91)
Bhutan	3.2 (1.6-5)	1.5 (0.7-2.3)	17.2 (9.8-26.2)	3.3 (1.9-5)	3.04 (2.95-3.12)
Bolivia (Plurinational State of)	77 (56.2-97.7)	2.5 (1.9-3.2)	445.3 (304.7-599.2)	5.3 (3.7-7.2)	2.43 (2.24-2.62)
Bosnia and Herzegovina	301.1 (282.8-320.3)	7.8 (7.3-8.3)	603 (482.1-747.2)	10.1 (8.1-12.5)	0.8 (0.71-0.9)
Botswana	20 (15.7-25.4)	3.8 (3.1-4.7)	97.2 (69.8-133.6)	8 (5.9-10.6)	2.2 (1.91-2.5)
Brazil	4552 (4350.9-4703.3)	5.5 (5.2-5.7)	14977.3 (13838.1-15869.4)	6.4 (5.9-6.8)	0.63 (0.57-0.7)
Brunei Darussalam	5.6 (4.7-6.6)	6.6 (5.6-7.7)	23 (19.8-26.3)	9.3 (8.1-10.6)	1.55 (1.38-1.73)
Bulgaria	854.9 (804.3-906.9)	6.9 (6.5-7.3)	1574 (1248.5-1961.6)	11.1 (8.8-13.9)	3 (2.51-3.49)
Burkina Faso	75.5 (58.3-96.7)	1.8 (1.4-2.3)	304 (230.3-386)	3.6 (2.8-4.6)	2.4 (2.34-2.46)
Burundi	54.2 (41.3-68.2)	2.4 (1.9-2.9)	98.6 (71.5-137.8)	2.3 (1.7-3.2)	-0.32 (-0.41--0.22)
Cabo Verde	2.9 (2.6-3.3)	1.2 (1.1-1.4)	44.2 (34.6-53.7)	10.8 (8.4-13.2)	6.64 (5.39-7.9)
Cambodia	95.6 (71-124.4)	2.2 (1.7-2.9)	418 (333.8-502.6)	3.7 (3-4.4)	1.92 (1.81-2.04)
Cameroon	161 (125.5-198.2)	3.9 (3-4.8)	866.4 (605.2-1190.7)	8 (5.7-10.8)	2.43 (2.32-2.54)
Canada	2775 (2633.4-2878.1)	8.6 (8.1-8.9)	6054.8 (5474.4-6647.1)	8.6 (7.8-9.4)	-0.01 (-0.08-0.06)
Central African Republic	24.5 (16.6-33.6)	2.2 (1.6-2.9)	45.6 (29.9-63.3)	2.2 (1.6-2.9)	0.03 (-0.02-0.08)
Chad	38.4 (29.9-48)	1.4 (1.1-1.8)	147.4 (117.1-184.9)	2.9 (2.3-3.6)	2.54 (2.38-2.7)
Chile	666 (623.2-710.7)	7 (6.5-7.5)	1873.6 (1689.1-2056.7)	7.8 (7-8.5)	0.42 (0.36-0.47)
China	27104.2 (23604.4-30844.3)	3.3 (2.9-3.8)	117374 (99862.7-136452.7)	6 (5.1-6.9)	2.25 (2.06-2.44)
Colombia	911.5 (864.4-957.2)	5.5 (5.2-5.8)	2495.5 (1919.6-3167.4)	4.7 (3.6-6)	-0.75 (-0.98--0.52)
Comoros	6.2 (3.7-7.9)	2.9 (1.8-3.7)	18.1 (14-23)	3.9 (3-4.9)	0.79 (0.65-0.92)
Congo	39.9 (26.9-54.2)	3.9 (2.8-5.1)	131.8 (84.4-191)	5.5 (3.6-7.7)	0.96 (0.72-1.2)
Cook Islands	0.5 (0.4-0.6)	3.8 (3.1-4.6)	1.2 (1-1.5)	4.9 (4.2-5.9)	0.62 (0.46-0.78)
Costa Rica	54.2 (49.4-59.1)	3.2 (2.9-3.5)	367.2 (284.6-461.4)	7.2 (5.6-9.1)	2.45 (1.9-3.01)
Croatia	530.6 (495.3-565.9)	8.4 (7.9-9)	813.2 (650.5-1019.2)	9.1 (7.2-11.4)	0.63 (0.46-0.8)
Cuba	178.6 (165.9-191)	1.7 (1.6-1.9)	1186.8 (967.4-1449.8)	6.2 (5-7.5)	4.29 (3.24-5.35)
Cyprus	33.2 (29.5-37.1)	4.3 (3.9-4.8)	156.7 (135.5-179)	8.2 (7.1-9.4)	2.8 (2.45-3.15)
Czechia	1644.4 (1574.7-1710.1)	11.9 (11.4-12.4)	2529.6 (2044.4-3053.5)	11.8 (9.6-14.3)	0.19 (0.02-0.36)
C�te d'Ivoire	103.2 (82.1-128.1)	2.8 (2.3-3.4)	518.2 (396.6-655.9)	5.5 (4.3-6.8)	1.97 (1.74-2.21)
Democratic People's Republic of Korea	473.2 (361.5-618.2)	3 (2.3-3.8)	1241.3 (941-1570.6)	3.9 (3-4.8)	1.03 (0.97-1.08)

Democratic Republic of the Congo	316.1 (247.9-402.9)	2.2 (1.7-2.8)	793.6 (600-1029.7)	2.4 (1.8-3.1)	-0.04 (-0.4-0.32)
Denmark	617.9 (582.3-648.3)	7.5 (7.1-7.8)	1193.6 (1061.2-1319.4)	10.1 (9-11.1)	0.91 (0.53-1.29)
Djibouti	3.3 (2.4-4.5)	2.6 (1.9-3.4)	23.5 (16.1-33.3)	4.3 (3.1-5.8)	1.8 (1.71-1.89)
Dominica	1.4 (1.2-1.5)	1.9 (1.7-2.1)	7.7 (6.2-9.4)	8.5 (6.8-10.4)	5.47 (4.52-6.42)
Dominican Republic	52.6 (46.1-60)	1.5 (1.3-1.7)	338.5 (231.6-458.9)	3.7 (2.6-5)	4.09 (3.6-4.59)
Ecuador	97.4 (91.1-104)	1.9 (1.8-2.1)	839.9 (669.5-1058.8)	5.8 (4.6-7.3)	3.55 (2.8-4.29)
Egypt	592.2 (534.5-642.6)	2.1 (1.9-2.3)	2768 (1899.5-3877.1)	4.4 (3.1-6.1)	2.43 (2.24-2.61)
El Salvador	50.9 (46.9-55.1)	1.8 (1.6-1.9)	314.7 (236.8-404.2)	5.3 (3.9-6.8)	3.89 (3.28-4.51)
Equatorial Guinea	3.7 (2.4-5.5)	1.9 (1.3-2.8)	27.4 (16.3-42)	6.4 (4-9.4)	4.94 (4.69-5.19)
Eritrea	17.4 (12.6-23.2)	1.8 (1.3-2.4)	77.3 (51.7-108.2)	3.1 (2.2-4.3)	1.59 (1.31-1.87)
Estonia	177.3 (165.4-189)	8.7 (8.1-9.2)	278 (218.4-346.9)	10.2 (8-12.9)	0.66 (0.42-0.91)
Eswatini	13 (9.7-17.1)	4.8 (3.7-6.3)	43.3 (29.3-60.3)	8.2 (5.7-11.2)	1.38 (0.71-2.06)
Ethiopia	233.8 (134.8-369)	1.3 (0.7-2)	602.5 (405.1-872.9)	1.6 (1.1-2.3)	0.81 (0.6-1.03)
Fiji	9 (7.3-10.9)	2.7 (2.2-3.3)	29.5 (23-37.1)	4.3 (3.4-5.4)	1.51 (1.44-1.58)
Finland	763.7 (724.4-796.7)	10.6 (10-11)	1377.7 (1219-1527.6)	10.7 (9.6-11.8)	0.26 (0.15-0.37)
France	6625.7 (6252.3-6907.9)	7.9 (7.5-8.2)	14045.5 (12223.6-15608.8)	9.8 (8.7-10.7)	0.91 (0.81-1.01)
Gabon	22 (16.9-34.4)	4.1 (3.2-6.2)	79.7 (54.1-111)	8.2 (5.7-11.2)	2.17 (2.02-2.33)
Gambia	5.2 (4-6.7)	1.6 (1.2-2)	31.5 (21.6-43.4)	3.5 (2.4-4.8)	2.59 (2.4-2.77)
Georgia	237.2 (193.5-306.6)	3.9 (3.2-5)	370.7 (306.1-437.9)	6.3 (5.2-7.5)	2.63 (2.05-3.22)
Germany	10605.7 (10037.3-11071.2)	8.2 (7.8-8.6)	21507.8 (19347.1-23692.6)	10.8 (9.9-11.8)	1.02 (0.81-1.23)
Ghana	270.9 (222.1-328.7)	4.6 (3.9-5.5)	1439.1 (1093.4-1884.2)	9.6 (7.3-12.6)	2.31 (2.18-2.44)
Greece	1432.5 (1358-1501.2)	9.4 (8.9-9.8)	2463.5 (2196.6-2699.3)	10 (9-10.8)	-0.03 (-0.13-0.06)
Greenland	5.1 (4.5-5.8)	16 (14.1-17.9)	12.8 (10.3-15.2)	19.3 (15.7-22.8)	0.31 (0.02-0.61)
Grenada	1.3 (1.2-1.5)	1.8 (1.6-2)	10.5 (9.2-11.9)	9.8 (8.6-11)	6.26 (5.16-7.37)
Guam	2.9 (2.5-3.3)	4.3 (3.6-5)	9.8 (8.1-11.7)	5.3 (4.4-6.3)	0.98 (0.75-1.2)
Guatemala	54.1 (47.1-62)	1.6 (1.4-1.8)	502.3 (391.3-630.9)	4.7 (3.7-5.9)	3.91 (3.08-4.75)
Guinea	38.1 (30.8-46.3)	1.2 (1-1.4)	99.4 (75.3-124.9)	1.9 (1.5-2.4)	1.61 (1.51-1.71)
Guinea-Bissau	10.5 (7.1-14.3)	2.7 (1.9-3.6)	31.5 (21-42.9)	4.7 (3.3-6.3)	2 (1.86-2.13)
Guyana	6.5 (5.6-7.5)	1.8 (1.6-2.1)	36.8 (28.1-46.8)	6.1 (4.7-7.7)	4.08 (3.24-4.92)
Haiti	38.7 (29.5-48.2)	1.3 (1-1.5)	165.5 (112.3-231.4)	2.5 (1.8-3.5)	2.71 (2.52-2.9)
Honduras	40.5 (32.4-47.5)	2.1 (1.7-2.5)	256.9 (155.5-369.9)	4.6 (2.8-6.5)	2.89 (2.74-3.04)
Hungary	1477.6 (1413.2-1547)	10.1 (9.7-10.6)	2331.2 (1922.7-2789.4)	12 (9.9-14.4)	0.61 (0.47-0.75)
Iceland	25.4 (23.2-27.7)	8.8 (8.1-9.6)	46.3 (39.9-52.8)	8.1 (7.1-9.2)	-0.27 (-0.39--0.15)
India	6168.7 (5023-7322.1)	1.5 (1.2-1.8)	33546.5 (29137.6-38690.4)	3.1 (2.7-3.6)	2.47 (2.33-2.6)
Indonesia	2180.7 (1803.9-2549.1)	2.4 (2-2.7)	10588.6 (6930.8-14830.9)	5.4 (3.5-7.5)	2.93 (2.88-2.98)
Iran (Islamic Republic of)	534.3 (433.6-645.5)	2.3 (1.8-2.7)	3360.7 (3124-3643.5)	4.9 (4.5-5.3)	2.81 (2.64-2.98)

Iraq	235.7 (163-318)	3.2 (2.2-4.3)	1329 (1007.4-1654.7)	6.1 (4.7-7.5)	2.56 (2.21-2.91)
Ireland	404.3 (382.9-426.3)	9.8 (9.3-10.4)	699.4 (617.6-778.5)	9.2 (8.1-10.2)	-0.21 (-0.27--0.15)
Israel	470 (441.3-497.8)	9.7 (9.1-10.3)	1273.1 (1135.3-1387.4)	10.7 (9.6-11.7)	0.2 (0.12-0.28)
Italy	8000.5 (7726.8-8186.2)	8.9 (8.6-9.1)	14418.8 (12787.7-15411.4)	9.6 (8.7-10.2)	0.24 (0.14-0.33)
Jamaica	25.1 (22.8-27.5)	1.4 (1.3-1.5)	154.5 (121.6-191.9)	5.1 (4-6.4)	4.3 (3.39-5.22)
Japan	15361.1 (14605.9-15775.1)	9.2 (8.7-9.4)	37462.4 (31495.5-40785.5)	9.6 (8.4-10.3)	0.34 (0.24-0.44)
Jordan	32.5 (27.3-38.9)	2.7 (2.3-3.2)	315.3 (262.1-374.2)	5.4 (4.5-6.4)	2.79 (2.62-2.96)
Kazakhstan	151.1 (135.5-172.8)	1.3 (1.1-1.4)	1129 (962.2-1311.6)	6.7 (5.7-7.7)	7.52 (6.41-8.65)
Kenya	114.8 (75.4-167.1)	1.5 (1-2.2)	695.7 (536.7-907.1)	3.4 (2.7-4.4)	3.3 (3.1-3.49)
Kiribati	0.8 (0.7-1)	2.4 (1.9-2.8)	1.8 (1.3-2.3)	3 (2.3-3.9)	0.33 (0.01-0.64)
Kuwait	21.3 (18.7-24.1)	4.1 (3.5-4.6)	121.1 (98.1-147.7)	5.6 (4.5-6.8)	1.68 (1.3-2.07)
Kyrgyzstan	113.3 (99.3-129.3)	3.8 (3.3-4.3)	232.2 (199.3-266.5)	5.3 (4.6-6.1)	1.27 (1.08-1.45)
Lao People's Democratic Republic	48.6 (34.3-68.2)	2.4 (1.7-3.3)	143.2 (111-182.3)	3.5 (2.8-4.4)	1.29 (1.2-1.38)
Latvia	292.4 (272.2-314.2)	8.2 (7.6-8.8)	411.1 (340.1-501.1)	10.2 (8.4-12.4)	0.51 (0.22-0.79)
Lebanon	71.5 (58.6-85.9)	3.3 (2.7-4)	342.3 (265.3-421.5)	6.6 (5.1-8.1)	2.79 (2.6-2.99)
Lesotho	25.4 (18.8-33.8)	2.7 (2.1-3.6)	70.9 (49.2-96.4)	6.1 (4.3-8.1)	2.96 (2.78-3.13)
Liberia	28.6 (22.9-35.6)	2.7 (2.1-3.3)	93.3 (66.2-124.3)	5.1 (3.7-6.7)	2.93 (2.53-3.33)
Libya	70.7 (44.7-98.1)	4 (2.5-5.6)	351.4 (268.9-453)	7.3 (5.6-9.3)	2.23 (2.08-2.38)
Lithuania	342.2 (322.3-362.2)	7.6 (7.1-8)	532.6 (434.8-650.8)	9.2 (7.4-11.3)	0.55 (0.32-0.78)
Luxembourg	54.4 (50.8-58.3)	9.9 (9.3-10.6)	93.4 (80.7-106.4)	9.1 (7.9-10.3)	-0.21 (-0.39--0.02)
Madagascar	98.5 (77.7-116.6)	2 (1.6-2.3)	245.4 (173.4-331.4)	2.4 (1.7-3.2)	0.55 (0.45-0.65)
Malawi	72.9 (60.6-86.2)	2.1 (1.7-2.4)	206.9 (159.2-261.6)	3.1 (2.4-3.9)	1.44 (1.34-1.55)
Malaysia	167.1 (142.4-195.1)	1.9 (1.6-2.3)	949.2 (736-1194.3)	3.8 (3-4.8)	2.44 (2.05-2.83)
Maldives	2.4 (1.6-3.4)	3 (2.2-4.1)	12.2 (10.1-14.6)	4.5 (3.7-5.4)	1.06 (0.89-1.24)
Mali	87.9 (74.8-103.3)	2.2 (1.9-2.6)	278.8 (208.3-364.5)	3.5 (2.6-4.4)	1.55 (1.5-1.59)
Malta	35.9 (32.5-39.2)	8.5 (7.7-9.2)	82.4 (69.4-95.6)	8.7 (7.3-10)	0.32 (0.18-0.46)
Marshall Islands	0.4 (0.3-0.4)	2.4 (2-2.9)	1.3 (0.9-1.7)	4 (3-5.2)	1.72 (1.64-1.81)
Mauritania	34.7 (25.6-45)	3.6 (2.7-4.6)	125.1 (90.4-164)	6.5 (4.8-8.3)	2.07 (2.01-2.13)
Mauritius	30.4 (28.2-32.8)	4.3 (4-4.6)	91.8 (73.5-112.4)	5.3 (4.3-6.5)	0.73 (0.55-0.92)
Mexico	2313.2 (2221.8-2381.5)	5.7 (5.5-5.9)	6853.3 (5866.5-7852.9)	6 (5.2-6.9)	-0.19 (-0.31--0.07)
Micronesia (Federated States of)	1.4 (1.1-1.8)	3.2 (2.6-4)	3.4 (2.4-4.8)	5.3 (3.7-7.1)	1.53 (1.29-1.77)
Monaco	9.2 (7.2-11)	12.5 (9.8-15.2)	17.9 (14.3-21.4)	17.8 (14.2-21.2)	1.44 (1.19-1.7)
Mongolia	32.2 (26-38.5)	3.2 (2.6-3.8)	137.5 (106-176.8)	6.3 (5-7.9)	2.28 (2.11-2.45)
Montenegro	44.7 (38.8-51.9)	7.3 (6.3-8.4)	107.7 (90.1-127.7)	10.9 (9.1-12.9)	1.71 (1.63-1.8)
Morocco	254.6 (205.1-309.3)	2 (1.6-2.4)	1167.8 (840.9-1516.9)	4 (2.8-5.2)	2.38 (2.17-2.6)
Mozambique	103.2 (85.5-121.8)	1.9 (1.6-2.3)	381.7 (265.6-545.6)	3.9 (2.7-5.5)	2.83 (2.69-2.98)

Myanmar	544.4 (393.9-717.6)	2.4 (1.8-3.2)	1721.7 (1348.7-2222.9)	4 (3.1-5.1)	1.85 (1.75-1.95)
Namibia	12.9 (10.4-15.6)	1.9 (1.5-2.3)	53.6 (41.6-69.3)	4.1 (3.2-5.2)	2.81 (2.68-2.94)
Nauru	0.1 (0.1-0.2)	4.4 (3.3-5.7)	0.2 (0.1-0.3)	6.1 (4.3-8.2)	0.73 (0.53-0.92)
Nepal	95.4 (53.1-142.8)	1.1 (0.6-1.7)	613.1 (367.6-897.9)	3 (1.8-4.4)	3.71 (3.43-4)
Netherlands	1773.6 (1677-1846.3)	8.8 (8.3-9.2)	3913 (3517.3-4307.1)	11.1 (10-12.2)	1.11 (0.94-1.29)
New Zealand	283.2 (265.5-301.6)	7.2 (6.7-7.6)	597.2 (539.9-653.5)	7.5 (6.8-8.1)	0.22 (0.08-0.37)
Nicaragua	35.4 (29.1-42.9)	2.5 (2-3.1)	238.5 (197.2-281.3)	6 (5-7)	2.99 (2.55-3.42)
Niger	38.1 (27.1-49.3)	1.5 (1-1.9)	164.6 (109.4-234.8)	2.4 (1.6-3.3)	1.67 (1.52-1.81)
Nigeria	880.2 (631-1143.2)	2.2 (1.6-2.8)	3217.3 (2468.3-4013.9)	4.3 (3.3-5.2)	2.4 (2.3-2.49)
Niue	0.1 (0.1-0.1)	3.9 (3-4.7)	0.1 (0.1-0.2)	6.4 (4.9-8.1)	1.66 (1.47-1.85)
North Macedonia	123.3 (113.4-134.3)	6.7 (6.2-7.3)	342.4 (272.2-429)	10.8 (8.6-13.4)	1.73 (1.59-1.86)
Northern Mariana Islands	0.5 (0.4-0.7)	3.6 (3-4.3)	3.7 (3.1-4.3)	7.8 (6.6-9)	3.39 (2.74-4.04)
Norway	605.3 (572.9-629)	8.6 (8.2-8.9)	896.8 (816.9-959.6)	9 (8.3-9.6)	0.34 (0.23-0.46)
Oman	12.8 (8.3-18.5)	2.2 (1.4-3.1)	85.9 (72.4-104.2)	6.2 (5.4-7.2)	4 (3.68-4.31)
Pakistan	858.9 (716.2-1005.8)	1.6 (1.3-1.9)	3100.9 (2403.8-3981.8)	3.1 (2.4-4)	2.39 (2.21-2.57)
Palau	0.8 (0.6-1)	8.4 (6.4-10.8)	2.2 (1.7-2.8)	12 (9.2-15)	1.06 (0.93-1.19)
Palestine	28.7 (18-41.4)	3.5 (2.2-5)	149.9 (125.5-178.5)	7 (5.8-8.3)	2.37 (2.12-2.62)
Panama	36.4 (33.1-40)	2.5 (2.3-2.8)	202.1 (154.5-259.8)	4.9 (3.7-6.3)	1.97 (1.7-2.25)
Papua New Guinea	21.7 (13.6-32.8)	1.3 (0.8-1.9)	78.8 (54.7-112.9)	1.8 (1.3-2.6)	1.15 (1.07-1.23)
Paraguay	48.8 (42.8-55.7)	2.3 (2-2.6)	335.4 (255.5-423.1)	6.3 (4.8-7.9)	3.56 (3.04-4.08)
Peru	237.8 (203.1-278.5)	2.1 (1.8-2.4)	1722.2 (1303.5-2249.9)	5.4 (4.1-7.1)	4.02 (3.28-4.77)
Philippines	958.7 (848.8-1078.2)	3.4 (3-3.8)	3249.6 (2654.3-3957.6)	4.4 (3.6-5.3)	0.53 (0.23-0.82)
Poland	4220.2 (4066-4343.7)	9.8 (9.4-10.1)	7446.9 (6268.6-8799.5)	10.6 (8.9-12.5)	0.1 (0.03-0.16)
Portugal	1027.3 (973.1-1080.9)	7.5 (7.1-7.9)	1842.5 (1627.2-2031.2)	7.4 (6.6-8.1)	0.05 (-0.02-0.12)
Puerto Rico	79.9 (73.4-86.3)	2.2 (2-2.4)	450.4 (346.3-574.2)	6.1 (4.7-7.8)	2.51 (1.7-3.33)
Qatar	4.2 (3.1-5.5)	4.9 (3.6-6.4)	46.7 (33.5-64.4)	8.1 (6.1-10.5)	1.9 (1.56-2.24)
Republic of Korea	2197.5 (2097.8-2321.3)	7.7 (7.3-8.2)	7303.1 (6519.3-8149.8)	8.2 (7.3-9.2)	-0.33 (-0.57--0.09)
Republic of Moldova	290.8 (267.4-318.5)	6.6 (6-7.2)	459.6 (397.4-529.8)	8 (6.9-9.1)	1.16 (0.65-1.66)
Romania	1832.8 (1749.2-1916.6)	6.5 (6.2-6.8)	3644.1 (2969-4465.4)	9.9 (8.1-12.2)	1.17 (0.99-1.36)
Russian Federation	13920.3 (13489.7-14482.3)	7.7 (7.4-8)	19377.9 (16968.1-21965.2)	8.2 (7.2-9.3)	-0.11 (-0.37-0.15)
Rwanda	85.6 (63-110.3)	3.1 (2.3-3.9)	198.9 (154.3-258.5)	3.6 (2.9-4.6)	0.18 (-0.05-0.41)
Saint Kitts and Nevis	0.8 (0.7-0.9)	2.1 (1.9-2.3)	5.8 (4.8-6.8)	9.5 (8-11.2)	5.16 (3.94-6.39)
Saint Lucia	1.7 (1.5-1.8)	2 (1.8-2.2)	16.3 (13.7-19.2)	7.7 (6.5-9.1)	4.11 (3.19-5.04)
Saint Vincent and the Grenadines	1.2 (1.1-1.3)	1.7 (1.6-1.9)	8.6 (7.4-9.8)	6.5 (5.6-7.4)	4.05 (3.4-4.7)
Samoa	3.6 (3-4.3)	4.3 (3.5-5.1)	6.2 (5-7.9)	4.4 (3.6-5.6)	-0.38 (-0.61--0.15)
San Marino	2.9 (2.5-3.4)	8.7 (7.4-10.1)	7.5 (5.1-10.6)	11.1 (7.4-16)	1.19 (1.07-1.32)

Sao Tome and Principe	0.9 (0.7-1)	1.4 (1.2-1.7)	3.1 (2.1-4.3)	3.1 (2.1-4.3)	2.78 (2.72-2.83)
Saudi Arabia	100.1 (67.2-139)	1.9 (1.2-2.6)	846.6 (655.8-1081)	5.1 (4.2-6.2)	2.84 (2.33-3.35)
Senegal	76 (61.7-88.8)	2.5 (2-2.9)	338.9 (273.4-417.8)	4.9 (4-6)	2.45 (2.27-2.62)
Serbia	764 (673.6-867.5)	6.9 (6-7.8)	1734.3 (1366.6-2156)	10.8 (8.5-13.4)	1.66 (1.52-1.81)
Seychelles	3 (2.7-3.4)	5.4 (4.8-6.1)	7.9 (6.9-9.1)	7.5 (6.5-8.6)	1.12 (0.97-1.26)
Sierra Leone	36.9 (29.3-45.3)	2 (1.6-2.4)	124.2 (97.6-159.3)	3.7 (3-4.7)	2.36 (2.3-2.43)
Singapore	113.3 (105.2-121.6)	5.4 (5-5.8)	425.3 (379.7-466.4)	5.6 (5-6.1)	0.33 (0.2-0.46)
Slovakia	507.3 (482.2-535.3)	8.5 (8.1-8.9)	964.5 (758.5-1207)	10.4 (8.2-13)	0.93 (0.77-1.09)
Slovenia	200.7 (156.6-255.7)	8.2 (6.4-10.5)	421.5 (329.8-541.4)	9.5 (7.4-12.2)	0.67 (0.47-0.86)
Solomon Islands	2.5 (1.8-3.5)	1.9 (1.4-2.5)	9.2 (6.1-12.6)	3.1 (2.2-4)	1.53 (1.27-1.78)
Somalia	44.5 (27.5-66.7)	1.8 (1.2-2.7)	107 (57.7-173)	1.7 (0.9-2.7)	-0.17 (-0.21--0.12)
South Africa	947.4 (794.4-1172)	4.8 (4-5.9)	2628.4 (2347.6-2955.5)	6.3 (5.6-7)	0.76 (0.46-1.06)
South Sudan	59.8 (43.2-88.6)	2.6 (1.9-3.9)	105.3 (73.3-151.8)	3 (2.1-4.2)	0.4 (0.35-0.44)
Spain	3827.7 (3617.5-4002.2)	7 (6.6-7.3)	7900.5 (6997.8-8687.9)	8 (7.2-8.8)	0.78 (0.62-0.94)
Sri Lanka	183.9 (165.7-202.9)	1.8 (1.6-2)	734.2 (548.5-969.9)	3 (2.2-3.9)	2.32 (2.14-2.51)
Sudan	125.4 (80.5-197.8)	1.4 (0.9-2.2)	654 (446.7-1014)	3.8 (2.6-5.8)	3.6 (3.25-3.96)
Suriname	4.3 (3.9-4.7)	1.7 (1.6-1.9)	42.7 (35.2-51.3)	7.3 (6-8.7)	4.74 (3.95-5.55)
Sweden	1559.5 (1449.6-1637.8)	10 (9.4-10.5)	1976.2 (1792.8-2128.6)	9 (8.3-9.7)	-0.65 (-0.91--0.4)
Switzerland	661.5 (616.2-699.5)	6.2 (5.8-6.6)	1581.1 (1405.9-1741.7)	8.7 (7.8-9.6)	1.11 (0.85-1.36)
Syrian Arab Republic	91.4 (67.6-118.6)	1.8 (1.3-2.4)	430.8 (319.3-587.7)	3.8 (2.8-5)	2.74 (2.55-2.92)
Taiwan (Province of China)	674.4 (647.7-702)	4.3 (4.1-4.5)	3386.9 (2637.7-4409.1)	8.5 (6.6-11.1)	2.51 (2.33-2.7)
Tajikistan	95.5 (65.6-142.2)	3.6 (2.4-5.4)	222.4 (182.1-274)	5.6 (4.7-6.9)	1.7 (1.42-1.98)
Thailand	1133.5 (1002.2-1286.4)	3.3 (3-3.8)	4140.5 (3096.4-5331.4)	4.1 (3.1-5.3)	0.01 (-0.27-0.3)
Timor-Leste	4.4 (2.9-6)	1.7 (1.1-2.3)	23.6 (16.9-30.7)	3.1 (2.2-4)	2.61 (2.26-2.96)
Togo	28.2 (22.5-34.7)	2.4 (2-3)	161.6 (116.9-217.3)	4.9 (3.6-6.5)	2.29 (2.16-2.42)
Tokelau	0 (0-0)	2.6 (2-3.4)	0.1 (0-0.1)	4.5 (3.1-5.8)	1.79 (1.69-1.89)
Tonga	1.6 (1.2-2)	3 (2.3-3.7)	3.5 (2.6-4.6)	4.5 (3.3-5.9)	0.97 (0.51-1.43)
Trinidad and Tobago	11.8 (10.8-12.9)	1.4 (1.3-1.6)	109.5 (82.8-142.4)	6 (4.6-7.8)	5.32 (4.22-6.43)
Tunisia	98.5 (75.4-121.8)	2.1 (1.6-2.6)	470.8 (350.2-623)	3.9 (2.9-5.1)	2.09 (2.06-2.13)
Turkey	1837.1 (1501.3-2261.5)	5.3 (4.3-6.5)	7132.4 (5728.7-8753.6)	8.2 (6.6-10.1)	1.83 (1.46-2.21)
Turkmenistan	25.7 (23.5-28.3)	1.4 (1.3-1.6)	141.4 (113.5-177.7)	3.9 (3.1-4.8)	3.66 (3.46-3.85)
Tuvalu	0.2 (0.1-0.2)	2.6 (2.1-3.2)	0.4 (0.3-0.5)	4 (3-5.4)	1.24 (1.06-1.41)
Uganda	152.2 (121.4-182.6)	2.5 (2-2.9)	660.9 (511.1-826.6)	5 (3.9-6.2)	2.53 (2.38-2.68)
Ukraine	3565.2 (2952.1-4230.3)	5 (4.1-5.9)	5948.6 (5022.4-7009)	8 (6.8-9.4)	1.26 (1.01-1.51)
United Arab Emirates	33 (18.3-58.5)	8.2 (4.3-14.6)	708.7 (358.8-1120)	17.6 (9.4-26.2)	2.67 (2.18-3.17)
United Kingdom	7966.6 (7631.1-8162)	8.6 (8.3-8.9)	12071.4 (11109.6-12692.1)	9.2 (8.6-9.7)	0.21 (0.16-0.26)

United Republic of Tanzania	284.7 (233.9-350)	2.8 (2.3-3.3)	892 (698.1-1151.8)	3.9 (3.1-4.9)	1.15 (1.07-1.24)
United States of America	28596 (27103.3-29427.7)	8.8 (8.4-9)	57487.9 (53665.2-60245.1)	10.1 (9.4-10.5)	0.49 (0.46-0.52)
United States Virgin Islands	3.1 (2.7-3.7)	3.9 (3.3-4.5)	20.7 (17.1-24.6)	11.3 (9.3-13.4)	4.18 (3.7-4.66)
Uruguay	412 (377.7-446.1)	10.5 (9.7-11.4)	811.5 (739.4-886)	14.5 (13.2-15.7)	1.11 (1.04-1.19)
Uzbekistan	169.5 (130.4-259.8)	1.5 (1.2-2.4)	843 (706-1004.8)	5.2 (4.4-6)	4.55 (4.31-4.78)
Vanuatu	1.2 (0.8-1.6)	1.9 (1.4-2.6)	5.1 (4-6.7)	3.1 (2.4-4)	1.59 (1.49-1.7)
Venezuela (Bolivarian Republic of)	145.1 (134.4-156.4)	1.6 (1.4-1.7)	1667.1 (1261-2156.9)	5.9 (4.4-7.5)	3.65 (2.66-4.64)
Viet Nam	705.8 (583.2-834.8)	1.8 (1.5-2.1)	3923.9 (3070-4959.7)	4.5 (3.6-5.7)	3.76 (3.54-3.98)
Yemen	58 (33.1-92.3)	1.2 (0.7-2)	277.5 (206.9-370.9)	2.2 (1.7-2.9)	2.46 (2.32-2.59)
Zambia	95.5 (77.1-116.4)	3.6 (2.9-4.3)	318.3 (225-443.9)	5.1 (3.7-6.9)	0.95 (0.84-1.06)
Zimbabwe	186 (162.8-213.3)	5.1 (4.4-5.7)	517.4 (397-658.6)	8.1 (6.2-10.2)	1.37 (1.08-1.66)
